# Overexpression of RPOTmp Being Targeted to Either Mitochondria or Chloroplasts in *Arabidopsis* Leads to Overall Transcriptome Changes and Faster Growth

**DOI:** 10.3390/ijms25158164

**Published:** 2024-07-26

**Authors:** Igor V. Gorbenko, Vladislav I. Tarasenko, Elena Y. Garnik, Tatiana V. Yakovleva, Alexander I. Katyshev, Vadim I. Belkov, Yuriy L. Orlov, Yuri M. Konstantinov, Milana V. Koulintchenko

**Affiliations:** 1 Siberian Institute of Plant Physiology and Biochemistry of Siberian Branch of Russian Academy of Sciences, Irkutsk 664033, Russia; vslav@inbox.ru (V.I.T.); tatyanpotapova@gmail.com (T.V.Y.); alex@sifibr.irk.ru (A.I.K.); yukon@sifibr.irk.ru (Y.M.K.); milana-koulintchenko@rambler.ru (M.V.K.); 2 The Digital Health Center, I.M. Sechenov First Moscow State Medical University of the Ministry of Health of the Russian Federation (Sechenov University), Moscow 119991, Russia; 3 Agrarian and Technological Institute, Peoples’ Friendship University of Russia, Moscow 117198, Russia; 4 Biosoil Department, Irkutsk State University, Irkutsk 664003, Russia; 5 Kazan Institute of Biochemistry and Biophysics of the Federal Research Center “Kazan Scientific Center of the Russian Academy of Sciences” (KIBB FRC KazSC RAS), Kazan 420111, Russia

**Keywords:** *Arabidopsis thaliana*, plant transcriptome, nuclear-encoded RNA polymerases (NEPs), organellar gene expression (OGE), anterograde/retrograde regulation, NAC transcription factors

## Abstract

The transcription of *Arabidopsis* organellar genes is performed by three nuclear-encoded RNA polymerases: RPOTm, RPOTmp, and RPOTp. The RPOTmp protein possesses ambiguous transit peptides, allowing participation in gene expression control in both mitochondria and chloroplasts, although its function in plastids is still under discussion. Here, we show that the overexpression of RPOTmp in *Arabidopsis*, targeted either to mitochondria or chloroplasts, disturbs the dormant seed state, and it causes the following effects: earlier germination, decreased ABA sensitivity, faster seedling growth, and earlier flowering. The germination of RPOTmp overexpressors is less sensitive to NaCl, while *rpotmp* knockout is highly vulnerable to salt stress. We found that mitochondrial dysfunction in the *rpotmp* mutant induces an unknown retrograde response pathway that bypasses AOX and ANAC017. Here, we show that RPOTmp transcribes the *accD*, *clpP*, and *rpoB* genes in plastids and up to 22 genes in mitochondria.

## 1. Introduction

Plant mitochondrial DNA is characterized by great variability in size (from 70 to 11,000 kb) and structure [1]. Typically, angiosperm mitogenomes contain 24 core genes and 17 variable genes [2] encoding tRNA, rRNA, ribosomal proteins, and subunits of respiratory complexes [3]. In addition to known genes, angiosperm mitogenomes also contain numerous open reading frames (ORFs), some of which have been shown to participate in establishing cytoplasmic male sterility (CMS) [4]. However, most mitochondrial ORFs are not conserved among angiosperms and are considered to be nonfunctional [5]. Plastid genomes vary in the range of 120–2000 kb and contain 80–100 genes [6] encoding housekeeping proteins and proteins of photosynthetic apparatus [7].

According to proteomic studies, the functions of mitochondria are performed by ~3000 different proteins, and this rises to up to 8000 proteins for plastids. Most of them (93–99%) are encoded in the nucleus, synthesized in cytoplasm, and imported into mitochondria and chloroplasts [8,9,10,11]. The ratio of the copy numbers of nuclear and organelle genomes in multicellular organisms can reach 1:5000. Plant organellar genomes are characterized by significant variation between the tissues and developmental stages of the plant. Plastid genome copy numbers can reach 50,000 per cell in the mesophilic cells of green leaves [12]. In plants, mitochondrial genome copy numbers are much lower than in animals and vary greatly; some mitochondria in somatic cells may have no DNA at all [13].

Regulation of the organelle gene expression (OGE) is crucial for plant growth and development. This type of regulation occurs mainly at the post-transcriptional level by control of translation, processing, splicing, decay, or RNA editing [14]. A coordinated expression of proteins encoded in both organellar and nuclear genomes provides the correct subunit ratio for proper complex assembly. In photosynthetic organisms, this regulation is complicated by cross-interactions of mitochondria and chloroplasts. Photosynthesis provides substrates for mitochondrial respiration but also depends on a range of compounds synthesized by mitochondria. Thus, these organelles are metabolically interdependent [15,16,17] and require intracellular mechanisms for a coordinated regulation of their functions. Consistent with this, dual localization is very common for mitochondrial and chloroplast proteins [18].

A detailed study of the regulation of proteins targeted to both chloroplast and mitochondria is thought to be a fruitful approach for unraveling complex interconnections between the retrograde pathways of these organelles [19,20]. It was shown that a decrease in prolyl-tRNA synthetase (PRORS1) content (dual-targeted protein in *Arabidopsis*) is associated with a partial loss of mitochondrial respiratory chain proteins and impaired protein synthesis in plastids [21]. The general suppression of nuclear photosynthetic genes has been shown to respond to disturbed mitochondrial and chloroplast translation, implying a synergistic role of the two organelles in the regulation of these genes.

Over 100 proteins have been reported to be dual-targeted to the mitochondria and chloroplasts in *Arabidopsis* [22,23,24]. Within the large group of dual-targeted soluble proteins, there is a strong bias toward proteins involved in nucleotide metabolism, DNA replication, recombination and repair, and tRNA biogenesis and translation [25]. Considering the fact that dual-targeted proteins are involved in organellar transcription, translation, proteolysis, and anti-oxidant defense and metabolism, it appears they are likely to impact many of the pathways activating retrograde signaling.

Mitochondrial and plastid gene expression systems are under the initial control of nuclear-encoded phage-type RNA polymerases (RPOTs), which trigger all transcription events in both organelles [19]. In a number of dicotyledonous plants, including *Arabidopsis*, the transcription of organellar genes is performed by three RPOTs, mitochondrial RPOTm, plastid RPOTp, and dual-targeted RPOTmp [26]. In mitochondria, RPOTm is the main RNA polymerase functioning at every stage of plant development, while RPOTmp transcribes only a few mitochondrial genes [26,27]. However, RPOTmp mitochondrial function is specific and important [27,28,29,30] since its loss leads to a deficiency of mitochondrial respiratory complexes and impaired plant development. RPOTmp transcribes genes that encode the subunits of complexes I (*nad1*, *nad2*, *nad4*, and *nad6*), IV (*cox1*), and a number of other mitochondrial genes, such as the *ccmC*, *rps4*, and *matR* genes [27]. RPOTmp absence leads to a significant decrease in the activity of respiratory complexes I and IV, delayed germination, slower growth, changes in leaf morphology, and late flowering [27,31].

In higher plants, two nuclear-encoded RNA polymerases (NEPs), RPOTp and RPOTmp, are both functional during the early stages of seed germination, i.e., when RPOTp plays the major role in chloroplast transcription [32]. Several plastid housekeeping genes (*rpoB*, *accD*, and *ycf2*) are transcribed by RPOTp [33], while the RPOTmp functions appear to be very limited and have been reliably shown to perform transcription only from the PC promoter of the *rrn16* operon [29]. As plants develop, NEP activity in plastids decreases, and photosynthesis-related genes became actively transcribed by plastid encoded RNA-polymerase (PEP) [32,34]. However, many plastid genes possess both PEP and NEP promoters [33,35].

Organellar gene expressions must be accurately regulated in their response to developmental and environmental changes. When plastid or mitochondrial gene expressions are impaired, a signal transmitted to the nucleus influences the expression of genes related to organelle functions. Thus, the nucleus controls the activity of organelles (anterograde control), and they, in turn, transmit information about their functional state to the nucleus (retrograde control), modulating nuclear gene expression. Reactive oxygen species (ROS) evidently trigger the mitochondrial retrograde response (MRR) in plants, which is most likely mediated by ANAC017 [36]. H_2_O_2_, which is the more stable and abundant ROS molecule, is a potential direct trigger of the retrograde response in both chloroplasts and mitochondria [36]. It is thought that H_2_O_2_ interacts directly with specific thiol-reactive redox proteins (including transcription factors) that regulate the expression of H_2_O_2_-sensitive stress-responsive genes [37]. OGE, along with the redox state of the organelles, ROS, and the metabolite content in organelles, is one of important sources of the retrograde signal to the nucleus [38,39].

Established tools to study the OGE role in retrograde responses include inhibitors of plastid translation (e.g., lincomycin) and line knockout for proteins involved in chloroplast or mitochondrial gene expression, mainly post-transcriptionally, such as PPR-proteins, mitoribosomal proteins, etc. RPOTmp is involved in both mitochondrial and plastid transcription, and we suggest RPOTmp-overexpressing plants could be an interesting tool to manipulate the gene transcription in both types of organelles and to study specific retrograde responses to changes in mitochondrial or plastid gene expression.

In this study, through using *Arabidopsis* plants that overexpress RPOTmp targeted to mitochondria or chloroplasts, as well as the *rpotmp* knockout line, we examined the effect of altered RNA polymerase transcript levels on plant transcriptomes, growth, and development. We show that an increased content of RPOTmp, regardless of its targeting, accelerates plant growth and disturbs the dormant seed state. We reported an increase in the transcript level of several mitochondrial and chloroplast genes in plants with elevated RPOTmp levels in mitochondria and chloroplast, respectively. An absence or overexpression of RPOTmp results in significant transcriptome changes that predominantly affect genes encoding mitochondrial proteins, the transcription factors of various families, and factors belonging to many metabolic pathways.

## 2. Results

### 2.1. Characteristics of the RPOTmp Overexpressors with the Mitochondrial or Chloroplast Targeting of Recombinant Proteins

**Figure 1:**
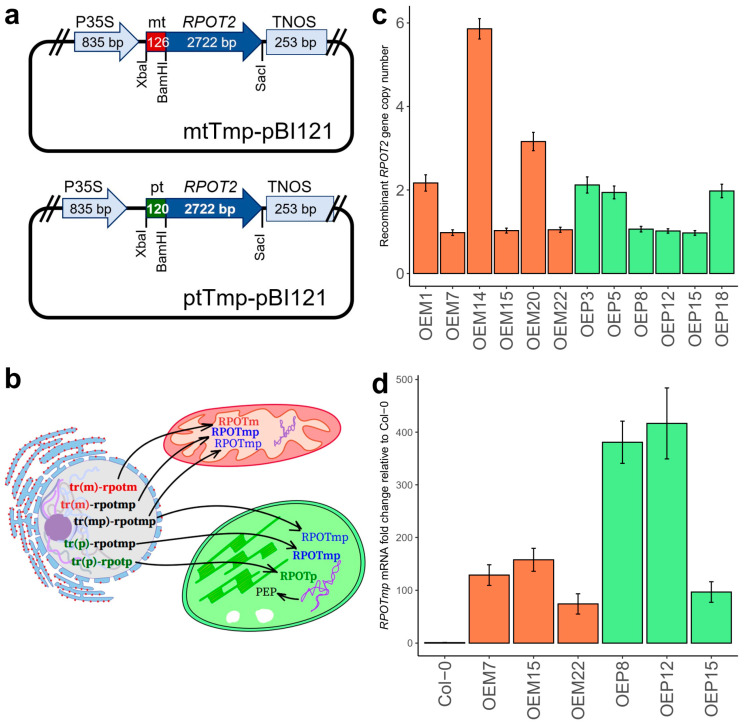
(**a**) Schematic representation of the genetic constructs used for obtaining transgenic lines [30]. Labels: the P35S-35S promoter of cauliflower mosaic virus, the TNOS-nopaline synthase terminator from the *Ti* plasmid of *Agrobacterium tumefaciens*, mt-cDNA of the transit peptide of *Arabidopsis* RPOTm, pt-cDNA of the transit peptide of *Arabidopsis* RPOTp, and RPOT2-cDNA of the *Arabidopsis* RPOTmp without transit peptide. (**b**) Schematic representation of the native and recombinant RPOTmp protein organellar localization in overexpressor plants. The tr(m), tr(p), and tr(mp)-sequences encoding the transit peptides of *Arabidopsis* RPOTm, RPOTp, and RPOTmp genes, respectively, where tr(mp)-rpotmp is the native *RPOT2*, and tr(m)-rpotmp and tr(p)-rpotmp are introduced transgenes. (**c**) The number of recombinant RPOTmp gene insertions in transgenic *Arabidopsis* lines with overexpression of RPOTmp targeted to mitochondria (OEM) or chloroplasts (OEP) determined by qPCR (*ACT7* gene copy number taken as 1). (**d**) The recombinant RPOTmp transcript levels in *Arabidopsis* transgenic lines determined by RT-qPCR (expression of *YLS8* was used for normalization); three or more biological replicates were used. In (**c**,**d**), the bar color represents mitochondrial (OEM, red) or chloroplast (OEP, green) targeting of recombinant RPOTmp.

Earlier we obtained two genetic constructs containing the catalytic part of the *Arabidopsis RPOTmp* gene combined with a transit peptide sequence from mitochondrial RNA polymerase RPOTm (mtTmp-pBI121) or chloroplast RNA polymerase RPOTp (ptTmp-pBI121) (Figure 1a) [30]. These genetic constructs were used for the agrobacterial transformation of wild-type *Arabidopsis*. The resulting variants of transgenic plants expressed the recombinant protein RPOTmp targeted to mitochondria (OEM) or chloroplasts (OEP) (Figure 1b).

We found that the obtained plant lines had significant diversity in the numbers of inserted transgene copies (Figure 1c). Subsequently, we used only lines containing a single copy of the transgene (OEM7, OEM15, OEM22, OEP8, OEP12, and OEP15) for most experiments. However, in several experiments where we tested the effects of stress conditions (darkness, ABA, sucrose, and NaCl), we used additional lines (OEM1, OEM14, and OEM20) that showed growth parameters close to the abovementioned OEM lines. According to RT-qPCR data, recombinant RPOTmp transcript levels in all these lines were orders of magnitude higher than the native RPOTmp mRNA level in Col-0 plants (Figure 1d).

**Figure 2:**
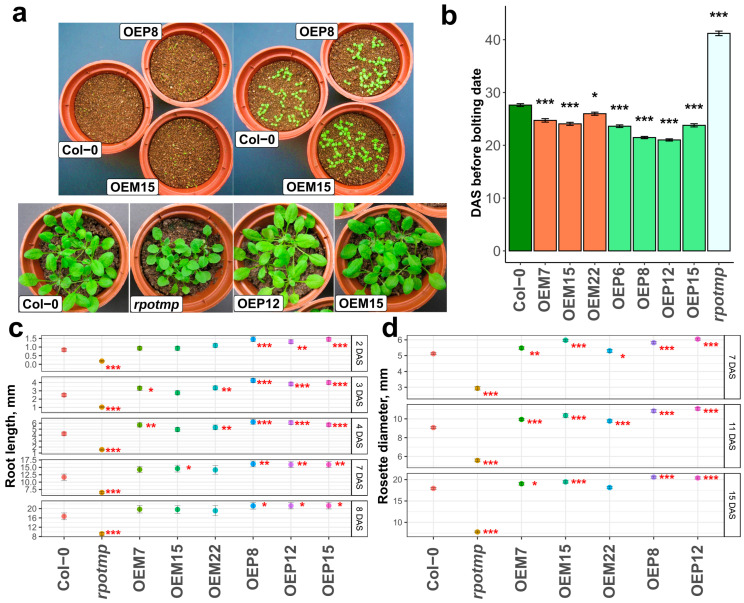
(**a**) Appearance of *Arabidopsis* wild-type plants (Col-0), *rpotmp* knockout line, and lines overexpressing RPOTmp in mitochondria (OEM15) or in chloroplast (OEP12). Left upper panel: 2-day-old seedlings; right upper panel: 7-day-old seedlings; bottom panel: 3-week-old seedlings. (**b**) Duration of the vegetative stage of development (from germination to flowering) of the studied *Arabidopsis* lines. (**c**) Root elongation in the RPOTmp overexpressors, Col-0, and the *rpotmp* lines during the first 8 days after the end of seed stratification. (**d**) Rosette diameter in the RPOTmp overexpressors, Col-0, and the *rpotmp* lines during the first 15 days after the end of seed stratification. The error bars represent standard deviations. Asterisks indicate differences from Col-0: *—*p* < 0.05, **—*p* < 0.01, and ***—*p* < 0.001. Sample size = 40 seedlings. DAS—days after stratification.

Rates of seed germination, growth, and development of both OEM and OEP plants were found to be accelerated in comparison to wild-type plants (Figure 2a). Moreover, the studied plant lines with RPOTmp overexpression were characterized by an earlier onset of flowering than the wild-type line (Figure 2b).

RPOTmp is known to be involved in the early stages of plant development [28,29]. To estimate a growth rate during the first days after stratification, two parameters were measured: primary root length (Figure 2c and Figure S1) and rosette diameter (Figure 2d). The *rpotmp* knockout plants had significantly shorter roots than the Col-0 plants on each day of observation. In overexpressor lines, there was a general trend toward an accelerated growth of primary roots compared to Col-0 seedlings, but only OEP lines showed significant differences from the wild type on each day of observation (Figure 2c and Figure S1). The same tendency was observed for a rosette diameter during the first 15 days after seed stratification (Figure 2d).

**Figure 3:**
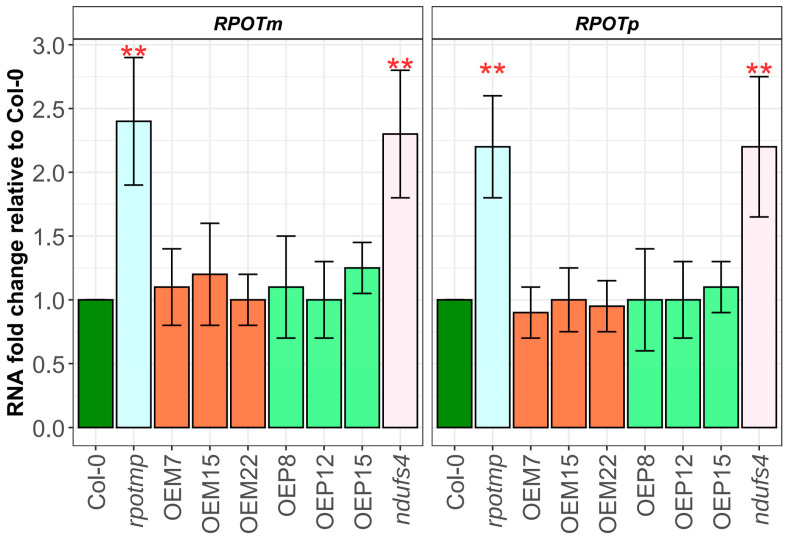
Transcript levels of the RPOTm and RPOTp genes in 12-day-old wild-type plants (Col-0) and the lines with overexpression of RPOTmp (OEM and OEP), as determined by RT-qPCR. The expression *YLS8* was used for normalization. Three or more biological replicates were used. Asterisks indicate differences from Col-0: **—*p* < 0.01.

We examined the effect of recombinant RPOTmp overexpression on the expression of two other phage-type RNA polymerases encoded by the nucleus: RPOTm and RPOTp (Figure 3). The transcript levels of RPOTm and RPOTp remained at the wild-type level in OEM and OEP plants (Figure 3) but increased 2–2.5-fold in the *rpotmp* line. To check whether the increased expression of these two RNA polymerases is associated with the absence of RPOTmp, we also used RNA from the complex I deficient *ndufs4 Arabidopsis* knockout line [40,41,42], which is phenotypically similar to the *rpotmp* mutant (delayed seed germination, growth, and development). Both RPOTm and RPOTp transcript levels were increased in *ndufs4* plants (Figure 3), indicating that the induction of these transcripts is associated with mitochondrial complex I deficiency rather than RPOTmp level alone.

### 2.2. Regulation of Seed Dormancy in RPOTmp Overexpressors Is Disturbed

We examined seed germination under both control and dark conditions (without a prior light stimulation). For the experiments, we used wild-type line Col-0, the *rpotmp* mutant line, overexpressor lines, and *abi4* mutant (abscisic acid insensitive 4, which is insensitive to ABA-mediated germination inhibition due to derepression of ABA catabolism [43], and it may show an easier release of seed dormancy [44]). The percentage of germinated seeds was estimated 6 days after the end of stratification. Plantlets of all studied lines grown in the dark had etiolated morphology (strongly elongated hypocotyls and unopened cotyledons without chlorophyll).

**Figure 4:**
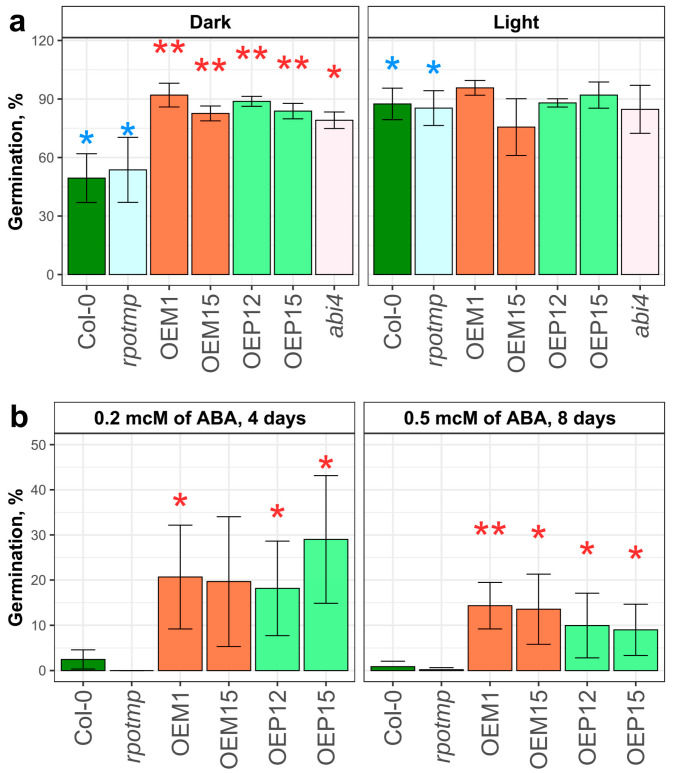
(**a**) Seed germination in the dark and in the light, 6 days after the end of seed stratification. *abi4*—abscisic acid insensitive mutant line. The asterisks above the diagrams indicate differences: blue—difference in the dark germination from the light one between same lines, red—difference in the germination from Col-0 in the same conditions: *—*p* < 0.05 and **—*p* < 0.01. (**b**) Seed germination in growth media containing 0.2 µM of ABA (4 days after the end of stratification) or 0.5 µM of ABA (8 days after the end of stratification). The error bars represent standard deviations. Red asterisks indicate differences from Col-0: *—*p* < 0.05 and **—*p* < 0.01. Sample size = 150.

Both the Col-0 and the *rpotmp* seeds had a lower percentage of germination in the dark than in the light (Figure 4a). Like the *abi4*, the OEM and OEP lines showed no significant differences between light and dark germination rates, but they did show a significantly easier release from dormancy in the dark compared to Col-0, suggesting that RPOTmp overexpression promotes some dysregulation of primary seed dormancy.

Sucrose can act as a negative regulator of development during seed germination. This is possibly due to the close relationship between sucrose signaling and ABA-mediated regulatory pathways [45]. Up to 3% of sucrose can be added to the growth medium to promote growth [46], while higher sucrose concentrations delay seed germination [47,48]. We examined the seed germination of Col-0, the *rpotmp* mutant line, and the RPOTmp overexpressor lines in growth media containing 0%, 6%, or 12% sucrose. In 12% sucrose, the *rpotmp* mutant seeds germinated much more poorly than the wild-type and the OEM or OEP lines (Figure S2a), and both OEM and OEP lines germinated better than the wild-type seeds in this medium. In the presence of 6% sucrose, a much earlier onset of germination of OEM and OEP lines was observed compared to Col-0 (Figure S2b). The obtained data indicate that RPOTmp overexpressors have a lower sensitivity to elevated sucrose content in a growth medium. Together with our data on dark-induced germination, it suggests a lower ABA sensitivity or an accelerated/derepressed ABA catabolism during seed imbibition and germination in RPOTmp overexpressor lines. To test this hypothesis, we placed the seeds on plates with additions of 0.2 or 0.5 μM of ABA to the growth medium. Exogenous ABA effected strong germination delay, but the seed germination of all studied overexpressor lines was dramatically higher than in the Col-0 and the *rpotmp* lines (Figure 4b).

### 2.3. Seed Germination of Arabidopsis Plants with Altered RPOTmp Expression in the Presence of NaCl

**Figure 5:**
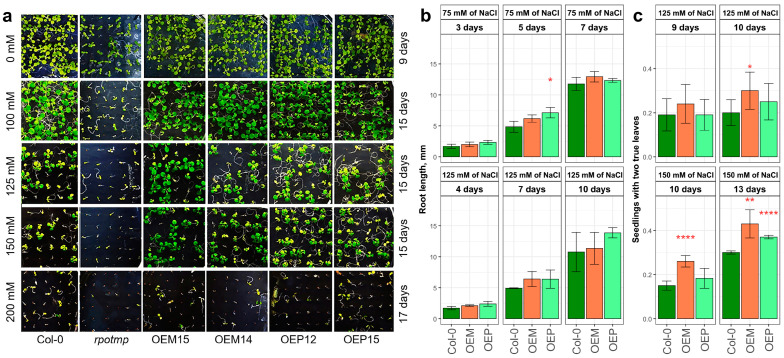
(**a**) Development of the Col-0, *rpotmp* mutant, and overexpressor seedlings in growth media containing 0, 100 mM, 125 mM, 150 mM, or 200 mM of NaCl. (**b**) Root lengths of the mitochondrial and plastid overexpressors grown in media with NaCl. (**c**) The number of seedlings with two true leaves under the same conditions. The error bars represent standard deviations. Asterisks indicate differences from Col-0: *—*p* < 0.05, **—*p* < 0.01, and ****—*p* < 0.0001.

ABA is involved in stress responses, so it is possible that stress conditions may differently affect the germination of wild-type seeds and lines with altered RPOTmp expression. We examined the seed germination and plant development in growth media with 0–200 mM of NaCl. While 100 mM of NaCl had virtually no visual effect on seed germination and leaf rosette development, 200 mM of NaCl had an almost lethal effect on all studied lines (Figure 5a). However, we observed that the RPOTmp overexpressor lines grew better compared to the Col-0 in media with 125–150 mM of NaCl (Figure 5a). The *rpotmp* knockout line showed very high sensitivity to NaCl (Figure S3).

We analyzed the phenotypic features of seedlings during the first two weeks of development: (1) root elongation on plates containing 75 and 125 mM of NaCl (Figure 5b), and (2) appearance of the first pair of true leaves on plates containing 125 and 150 mM of NaCl (Figure 5c). The root elongation was inhibited with 125 mM of NaCl compared to the growth with 75 mM of NaCl. The roots of OEM and OEP lines have a tendency to elongate faster than Col-0 roots during the first week under both NaCl concentrations (Figure 5b). The number of seedlings with the first pair of true leaves developed was estimated as a percentage to all germinated seeds on 125 mM and 150 mM of NaCl for several days. The OEM lines demonstrated the highest number of seedlings with their first pair of true leaves developed (Figure 5c), while seedlings of the *rpotmp* mutant line did not reach this stage of development and mostly died in these media.

### 2.4. Microarray Analysis of the Transcriptome of Arabidopsis Lines with Altered Expression of RPOTmp Targeted to Mitochondria or Chloroplasts

**Figure 6:**
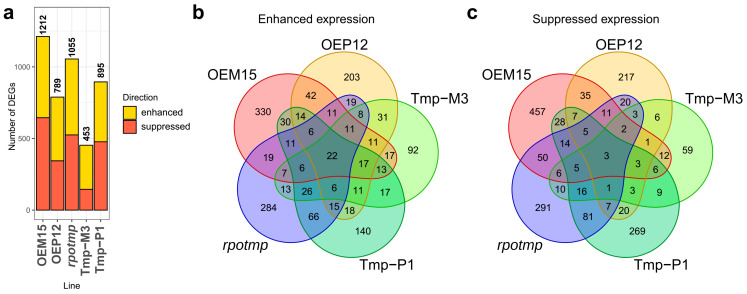
(**a**) The total number of microarray probes that were differentially expressed compared to wild-type plants. (**b**,**c**) Venn diagrams of DEGs with enhanced and suppressed expression.

Using the DNA microarray method, we performed a genome-wide analysis of transcriptome changes in the *rpotmp* mutant line, as well as in the lines overexpressing or complementing RPOTmp in mitochondria (OEM15 and Tmp-M3) or in chloroplast (OEP12 and Tmp-P1), in comparison to wild-type plants. OEP12 and OEM15 were selected for microarray analysis because they demonstrated the highest level of recombinant RPOTmp transcripts, as well as a single copy of transgene insertion. To determine genes with differential expressions in relation to the wild-type plants, we used an integrated approach that takes into account the log fold changes (LFC) of gene transcript levels and *p*-values (moderated *t*-test). Figure 6a shows the DEG numbers detected by this method (LFC > 1 at *p* < 0.2, the tabular data of which are shown in Data S1) as well as Venn diagrams (Figure 6b,c). The total number of differential expressed genes in all studied lines was 3141 (Data S1).

The OEM15 line had the largest number of DEGs (Figure 6a), as well as unique DEGs with enhanced and suppressed expression (330 and 457, respectively) (Figure 6b,c). The complementation of the Tmp-M3 line had the lowest number of DEGs, as well as the lowest number of unique DEGs with increased and decreased expression (92 enhanced and 59 suppressed). In addition, it had 145 DEGs (99 enhanced and 46 suppressed) in common with the *rpotmp* line. A transgenic line with a complementation of RPOTmp functions in chloroplasts, i.e., Tmp-P1, had 290 unique DEGs (158 enhanced and 132 suppressed). The complementation of RPOTmp functions in mitochondria caused a larger decrease in the number of DEGs than complementation in chloroplasts; this was most likely due to the fact that the main function of the RPOTmp protein was fulfilled in mitochondria. Considering the large number of unique DEGs in the OEM15 (330 enhanced and 457 suppressed) and OEP12 (203 enhanced and 217 suppressed) overexpressor lines, they was only 201 (134 enhanced and 67 suppressed) common DEGs found (Figure 6c). This indicates that pattern of expression changes in response to the overexpression of RPOTmp is more specific to the targeted organelle rather than being common for all overexpressor lines.

**Figure 7:**
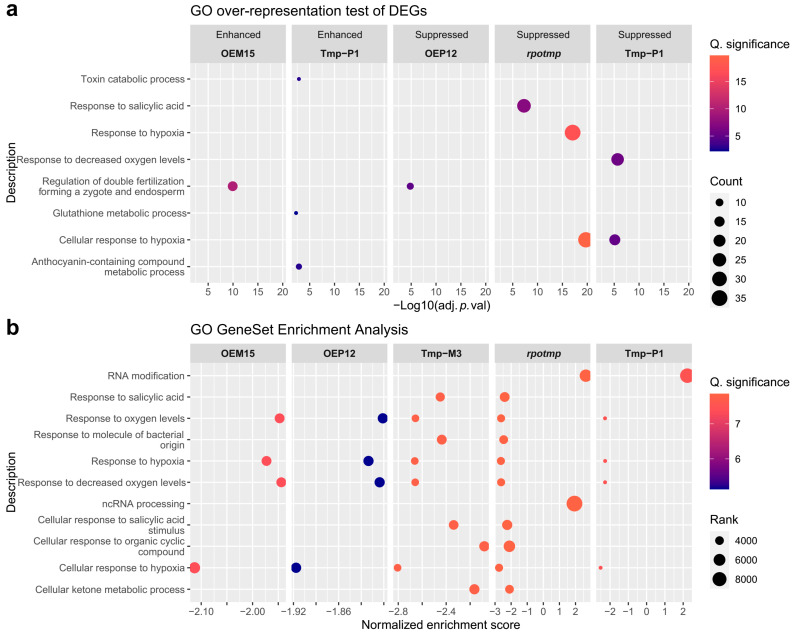
(**a**) Gene ontology over-representation analysis of DEG results and (**b**) GO gene set enrichment analysis results of the full transcriptome gene set. *p*-values were calculated by the Benjamini–Hochberg method.

To characterize the transcriptome changes in the studied lines, we conducted Gene Ontology Biological Process (GO BP) overrepresentation (ORA) and gene set enrichment (GSEA) analyses (Figure 7). The regulation of double fertilization forming a zygote and endosperm term was enriched in the enhanced DEGs of OEM15 and in the suppressed DEGs of OEP12. Suppressed DEGs of lines lacking RPOTmp in mitochondria (*rpotmp* and Tmp-P1) were enriched with terms associated with Hypoxia, and only the suppressed DEGs of *rpotmp* were enriched with Response to Salicylic Acid terms. Gene set enrichment analysis of the full transcriptome data showed that all studied lines had suppressed hypoxia-related processes (Figure 7b). The GSEA of the GO Cellular Component showed the suppressed term Nucleolus (GO:0005730) in OEM15 and OEP12 and the enriched term Preribosome (GO:0030684) in *rpotmp* and Tmp-P1.

In the work of van Aken et al., several genes were identified as marker genes for organellar disorders [49]. We found that the expression of the AT12CYS-2 protein (located in mitochondria [50] and is the marker gene of mitochondrial disorders [49]) was highly increased (LFC ˃ 3) in the *rpotmp* mutant and complementation lines (Tmp-M3 and Tmp-P1). The transcript levels of the two proteins known to be involved in mitochondrial retrograde response (MRR)—HRG1 (At2g41730) and UPOX1 (At2g21640)—were increased in the *rpotmp* and Tmp-P1 lines. No increase in the expression of any marker gene of disorders was found in the OEM15 line (Figure S4).

It was previously shown that the transcript levels of subgroup E PPR proteins, which are presumably involved in organellar RNA editing under stress conditions, vary at different stages of plant development and under abiotic stress [51]. In our analysis, among the 105 PPR proteins of the E and E+ subclasses, only 5 ones were presented among the DEGs in at least one of the studied lines (Figure S5). The expressions of specific genes are shown in Data S5.

### 2.5. Influence of the RPOTmp Altered Expression on Mitochondria Functions

**Figure 8:**
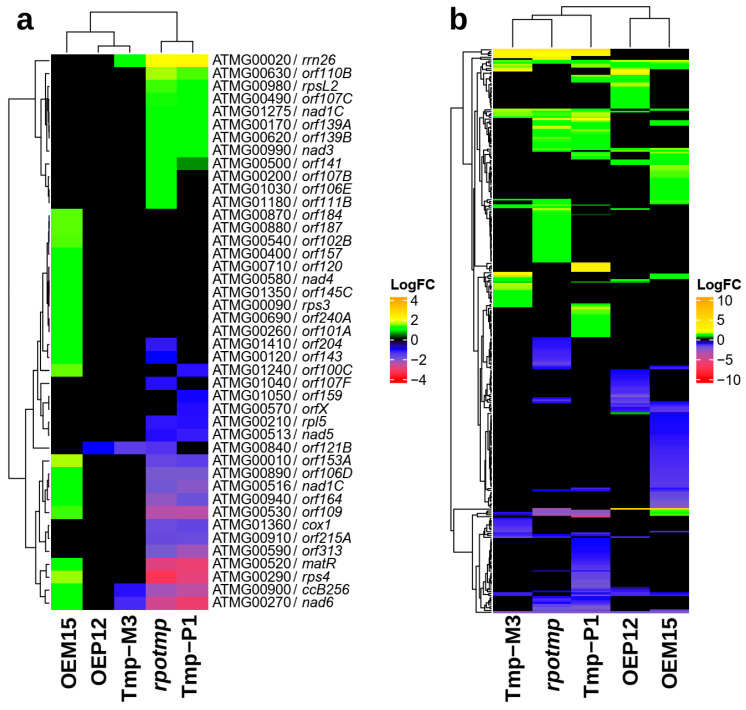
(**a**) Differentially expressed mtDNA-encoded genes and (**b**) differentially expressed nuclear-encoded genes with mitochondrial targeting, as determined by the DNA microarray method.

Using the DNA microarray method, we detected DEGs encoding proteins with mitochondrial localization that are encoded by mitochondrial (Figure 8a) or nuclear genomes Figure 8b).

The OEM15 line was characterized by an enhanced expression level of 22 mitochondrial transcripts (including 15 open reading frames of unknown function), which were suppressed or unaffected in the *rpotmp* line (Figure 8a).

The expression profile of mitogenome-encoded DEGs in the complementation line of Tmp-P1 was very similar to the profile of the *rpotmp* mutant. Conversely, in the complementation Tmp-M3 line, the expression profile was restored close to the wild-type level (Figure 8a), suggesting a specific involvement of RPOTmp in mitochondrial gene expressions. The low number of detected DEGs in the Tmp-M3 line was probably due to the fact that the recombinant RPOTmp transcript levels in this line was much lower than in OEM15 [30], making the molecular phenotype of Tmp-M3 closer to the Col-0 rather than the overexpressor lines.

**Figure 9:**
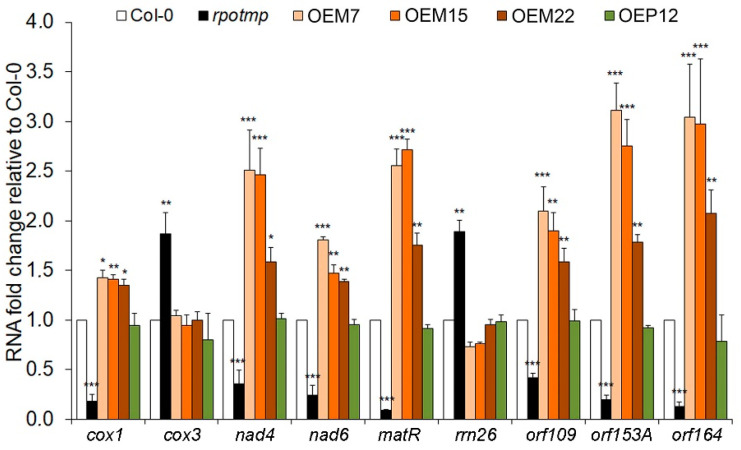
mRNA fold change in several mitochondrial genes in the rpotmp mutant line and lines with RPOTmp overexpression compared to the wild-type (Col-0) plants. RNA was isolated from 12-day-old seedlings. The average values with standard deviations of three biological replicates are shown, where *, **, and *** are statistically significant differences at *p* ≤ 0.05, *p* ≤ 0.01, and *p* ≤ 0.001, respectively.

In addition to DNA microarray expression analysis, we analyzed mtDNA gene expressions by RT-qPCR (Figure 9) using the RNA isolated from the 12-day-old seedlings.

In agreement with previous studies, genes encoding respiratory complex IV subunits changed their expression in the *rpotmp* line: the expression of *cox1* was decreased, and the expression of *cox3* was increased. The *cox3* transcript level remained unchanged in all lines with RPOTmp overexpression, and the *cox1* transcript level increased only in OEM lines. Similarly, the transcript levels of the respiratory complex I subunits *nad4* and *nad6* were decreased in the *rpotmp* line and increased in the OEM lines (Figure 9). The levels of the *matR* gene (RNA processing in mitochondria) and three ORF transcripts (*orf109*, *orf153A*, and *orf164*) were suppressed in the *rpotmp* line and enhanced in the OEM lines (Figure 9). We assume that mitochondrial genes with enhanced expressions in the OEM lines are transcribed by RPOTmp. The increase in some transcript levels in the *rpotmp* line was assumed to occur due to the increased copy number of the mitochondrial genome, which was approximately three times higher than in the wild-type plants [27,30]. In the DNA isolated from seedlings of all the studied OEM and OEP lines, the copy number of two mitochondrial genes, *cox1* and *nad3*, remained close to that in the wild-type plants (Figure S6).

Differential expression was found for 301 nuclear genes encoding mitochondrial proteins (GO:0005739) in at least one of the studied lines (Figure S7, Data S4). In the OEM15 line, the expression of 64 genes encoding mt-proteins was downregulated, while the expression of 46 genes was upregulated. The findings on particular gene expressions are summarized in Data S3.

To detect the genes probably involved in retrograde regulation in response to the altered RPOTmp level in mitochondria, we investigated several groups of DEGs: the retrograde regulated genes (RRG) in response to the increased content of RPOTmp in mitochondria were detected as DEGs of the OEM15 line, which were not present in the DEGs of the OEP12, Tmp-P1, and the *rpotmp* mutant lines. We found enhanced expressions of 286 (enriched in the Regulation of fertilization GO term; Figure S8) and suppressed expression of 418 genes. Among the found genes, 22 genes with enhanced and 22 genes with suppressed expressions encoded proteins with mitochondrial localization (Data S2).

The RRGs in response to the absence of RPOTmp in mitochondria were detected as DEGs shared between *rpotmp* and Tmp-P1, but these were not present among the DEGs of OEP12 and OEM15: 90 genes were upregulated (enriched GO terms of Mitochondrial organization and transport) and 59 were downregulated (enriched GO terms of Response to hypoxia, salicylate, ROS, and heat; Data S2, Figure S8). Enhanced expressions of 22 genes encoding proteins with mitochondrial localization was found.

**Figure 10:**
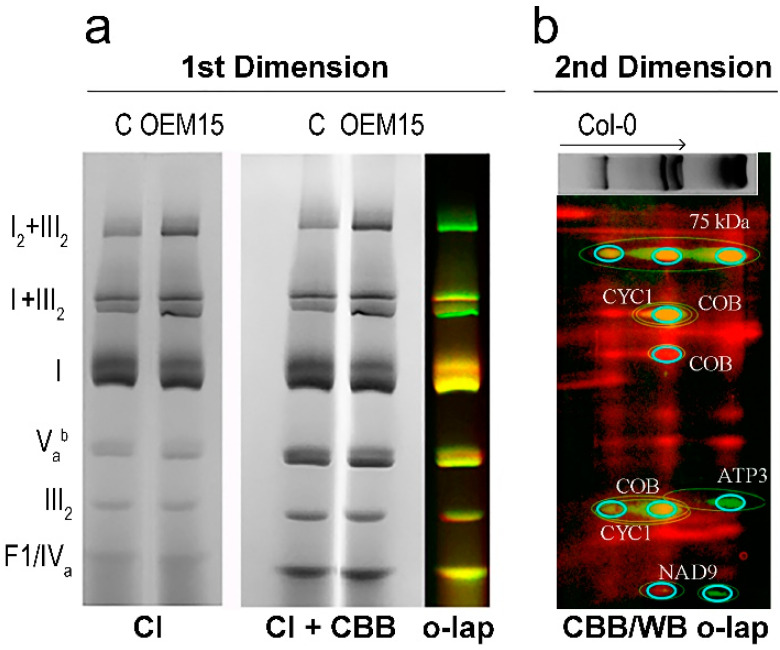
(**a**) BN-PAGE of *Arabidopsis* mitochondria. C—Col-0 wild-type plants, CI—complex I in-gel activity, CBB—Coomassie brilliant blue, and o-lap—overlap of electrophoregrams (Col-0 red and OEM15 green channel). (**b**) Overlapped image of the 2D BN/SDS-PAGE of Col-0 mitochondria and Western blotting. The direction of the first dimension is marked by an arrow.

To study the possible effects of altered expression levels of genes encoding respiratory complex subunits in lines with RPOTmp overexpression, we analyzed the supramolecular organization of the electron transport chain (ETC) using the BN-PAGE method (Figure 10). The analysis revealed a slight increase in the number and activity of I+III_2_ and I_2_+III_2_ supercomplexes (the composition was confirmed using immunoblotting; Figure 10b), but not in the activity of free complex I in the OEM15 line compared to the Col-0 line (Figure 10a). It is possible that an increased number of complex I in the OEM15 mitochondria leads to an enhanced formation of supercomplexes. There was no difference in the activity of complex IV (Figure S9) between the Col-0 and the Col-M15 lines.

### 2.6. Influence of the RPOTmp Altered Expression on Chloroplast Functions

Our microarray data revealed 271 nuclear genes encoding proteins with chloroplast localization (GO:0009507) among the DEGs (Figure S10, Data S4, and the findings on particular gene expressions are summarized in Data S3). To identify genes that may mediate retrograde regulation in response to increased RPOTmp levels in chloroplasts, we selected the DEGs of OEP12 that were not presented in the DEGs of OEM15, *rpotmp*, and Tmp-M3. A total of 8 nuclear genes encoding chloroplast proteins were upregulated, and 11 genes were downregulated only in the OEP12 line. For RRG in response to the absence of RPOTmp in chloroplasts, we selected the shared DEGs of Tmp-M3 and *rpotmp* that were absent from the OEM15 and OEP12 DEGs: 36 genes were upregulated and 16 were downregulated (enriched GO terms of Response to salicylate, hydrogen peroxide, and absence of light were found; Figure S8, Data S2). In response to the absence of RPOTmp in chloroplasts, an increase in the expression of two genes and suppression of one gene encoding proteins with plastid localization were observed in the *rpotmp* and the Tmp-M3 lines (Data S2).

The established function of RPOTmp in *Arabidopsis* proplastids is associated with transcription of the *rrn16* operon from the PC-promoter at stages of seed imbibition and germination [29,52]. Swiatecka-Hagenbruch et al. [53] showed that, in addition to the PC promoter, RPOTmp is also capable of initiating transcription from the PclpP-58 NEP promoter. However, Kühn et al. [27] did not find any changes in the steady-state levels of chloroplast transcripts in the *rpotmp* knockout line.

**Figure 11:**
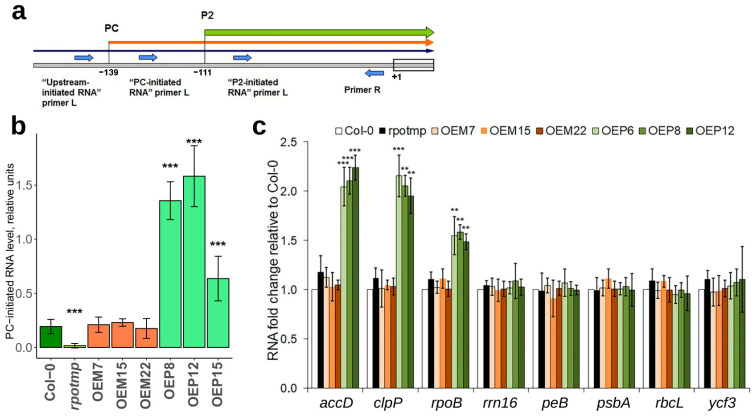
(**a**) Schematic representation of the *rrn* operon transcription initiation sites in *Arabidopsis* and the sets of primers used to evaluate the levels of RNA precursors. Black arrow represents upstream-initiated RNA, orange—PC-initiated RNA, green—P2-initiated RNA. (**b**) The transcript levels of *rrn16* initiated from the PC promoter in germinating seeds of the studied lines. (**c**) Transcript levels of several chloroplast genes in the studied lines. The average values of three biological replicates with standard deviations are shown. ** and *** are statistically significant differences at *p* ≤ 0.01 and *p* ≤ 0.001, respectively.

To analyze the expression of *rrn16* from the PC promoter, we used a set of primers [30] to amplify the 5′ region of the *rrn16* operon to detect RNA precursors by RT-qPCR (Figure 11a). RNA from the 12-day-old seedlings of OEM and OEP lines was used. Increased transcript levels were detected in all of the OEP lines (Figure 11b).

Next, we used RT-qPCR to analyze the transcript levels of several chloroplast genes transcribed either exclusively from PEP- (*petB* and *psbA*) or NEP- (*accD* and *clpP*) promoters, or from both PEP- and NEP-promoters (*rbcL*, *rpoB*, *rrn16*, and *ycf3*). A specific increase in the *accD*, *clpP*, and *rpoB* transcript levels was detected in seedlings of OEP lines (Figure 11c).

### 2.7. Transcription Factor Expression in Lines with RPOTmp Overexpression

A total of 182 transcription factors was found among the DEGs of the studied lines (Figure S11, Data S5). The factors of 31 families were presented among the DEGs, among which the MYB domain TF family was the most represented (25 factors). Other families included 17 ethylene response factors, 15 NAC-domain factors, 13 factors with basic “helix-loop-helix” motifs, 16 MADS-box domains, 10 factors of the LBD family, 7 of the WRKY family, and 79 TFs of other groups. The detected TFs were overrepresented with Response to hypoxia (31 genes), Floral organ development (11 genes), Pollen development (10 genes), Response to ethylene (8 genes), and Response to gibberellin (7 genes) GO terms. The largest number of unique TF DEGs was found in the OEM15 line (16 enhanced and 33 suppressed).

The expression of several MYB-like transcription factors known to be involved in the water stress response via regulation of the stomatal movement, which is in control of the suberin and cuticular wax syntheses and in regulation of flower development [54], was modulated in the lines with RPOTmp overexpression. The findings on particular gene expressions are summarized in Data S3.

The transcript levels of several genes of the NAC (no apical meristem) TF family were observed to respond to the absence of RPOTmp in mitochondria (Data S1). Using RT-qPCR analysis, we examined the expression of some NAC domain transcription factors in studied plant lines. ANAC017 (At1g34190) and ANAC013 (At1g32870) play important roles in the MRR associated with the oxidative stress response, as well as in developmental regulation [55,56]. ANAC044 is a known mediator of the DNA damage response in plants [57].

**Figure 12:**
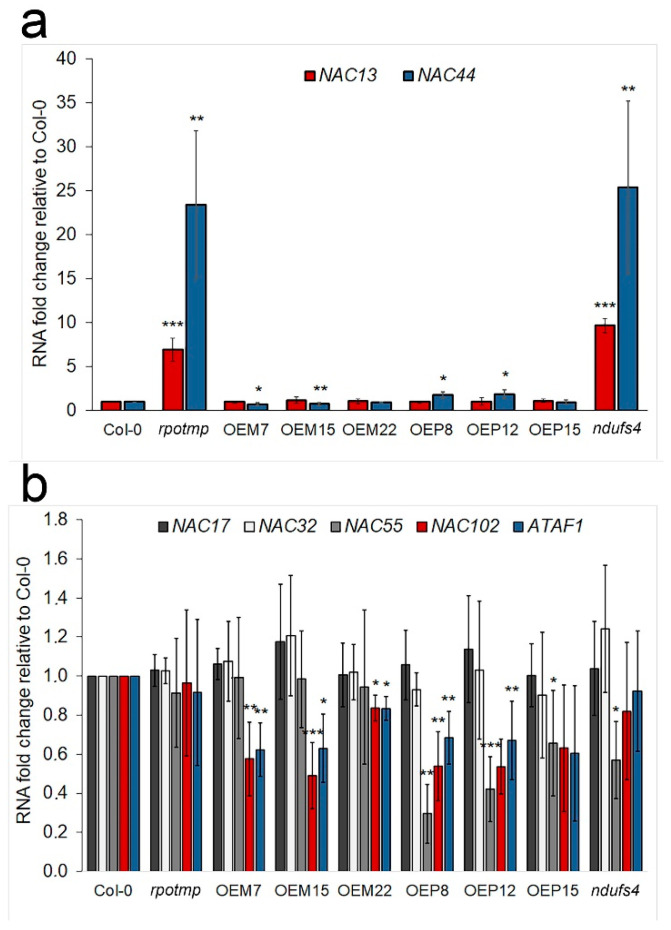
The transcript levels of NAC transcription factors in 12-day-old *rpotmp* and *ndufs4* mutant lines, as well as in lines with RPOTmp overexpression, as determined by RT-qPCR. (**a**) The relative mRNA levels of ANAC013 and ANAC044 in the studied lines, and (**b**) the relative mRNA levels of ANAC017, ANAC032, ANAC055, ANAC102, and ATAF1 (ANAC002) in the studied lines. *YLS8* was used for normalization. The average values of three biological replicates with standard deviations are shown. *, **, and *** are statistically significant differences at *p* ≤ 0.05, *p* ≤ 0.01, and *p* ≤ 0.001, respectively.

An increase in the expression of *ANAC013* and *ANAC044* was found in both the *rpotmp* and the *ndufs4* mutants (Figure 12a); meanwhile, the *ANAC044* expression was reduced in OEM7 and OEM15, while it was slightly increased in the OEP8 and OEP12 lines.

ANAC002/ATAF1 (At1g01720), ANAC102 (At5g63790), ANAC032 (At1g77450), and ANAC055 (At3g15500) are members of the A subfamily of stress-responsive NAC (SNAC-A) transcription factors, the expression of which is induced by abiotic stresses and is mediated by ABA [58]. The transcript levels of ATAF1 and ANAC102 were decreased in all the lines with overexpression of RPOTmp, regardless of recombinant RPOTmp targeting (Figure 12b). The expression of ANAC055 was decreased in the OEP lines and in the *ndufs4* mutant line (Figure 12b).

## 3. Discussion

### 3.1. Overexpression of Arabidopsis RPOTmp Disturbs the Dormant State of Seeds and Accelerates Seedling Growth

Mitochondrial biogenesis is extremely important for energy production during the early stages of seed germination [10,59,60,61]. The RPOTmp knockout leading to a significant change in the expression of a number of subunits of respiratory complexes I (*nad1*, *nad4*, *nad5*, and *nad6*) and IV (*cox1*) results in a decrease in the activity of these complexes [27]. Alternative NADH dehydrogenases support plant cellular respiration in complex I-deficient plants, although the physiology, development, and fertility in such plants are severely impaired [42,62]. For the *rpotmp* knockout plant, delayed germination, slowed root elongation, reduced leaf size, and delayed flowering were observed [27]. The lines overexpressing RPOTmp in mitochondria (OEM) and in chloroplasts (OEP) exhibited slightly earlier seed germination, faster root elongation during the first days of development, slightly larger leaf rosettes, and earlier flowering (Figure 2). Faster root elongation and earlier bolting dates were more manifested in the OEP lines. Our tests for dark germination, germination in the presence of elevated sucrose concentration, and germination with exogenous ABA added results with even clearer differences between the RPOTmp overexpressors and wild-type plants during early development stages (Figure 4 and Figure S2).

The primary seed dormancy depth determines the timing of germination and depends on the balance of ABA and gibberellin (GA). Seed imbibition causes ABA degradation, and then GA-mediated activation of germination occurs [63]. Several transcription factors oppositely regulate the ABA and GA metabolism pathways [64]. ABI4 is a positive regulator of the dormancy state during seed development and a repressor of ABA catabolism. The reduced ABA content and increased GA level in the dry seeds of the *Arabidopsis abi4* mutant compared to the wild type [44] may cause faster germination of these seeds when without light stimulation in our experiments. We observed that RPOTmp overexpression led to a reduction in the dormancy depth to the same extent as ABI4 transcription factor absence in the *abi4* mutant line (Figure 4a). Similar sensitivity disorders have been described for a number of *Arabidopsis* mutants carrying defects in genes encoding various elements of ABA-mediated regulatory pathways [65].

During seed germination, elevated sucrose concentration can inhibit seed development, leading to a total arrest of germination in some cases. This is possibly due to the interconnections of sucrose signaling and ABA-mediated regulatory pathways [66]—elevated sucrose content leads to increased ABA content [67]. Germination of the complex I-deficient *ndufs4* mutant was inhibited by 3% sucrose in the growth medium, and the addition of GA completely relieved the inhibition [40]. The authors suggest that the decreased rate of ATP synthesis in the *ndufs4* mutant caused slower germination and an increased ABA level. It is possible that a decrease in the activity of respiratory complexes in the *rpotmp* line leads to a decrease in ATP content, which may cause similar negative effects on germination and increase sensitivity to ABA, as was observed in the *ndufs4* mutant line. In a recent study, Bychkov et al. [68] showed that the *rpotmp* mutant line is more sensitive to ABA treatment in root elongation and germination assays.

The absence of functional RPOTmp strongly affects plant development in media with added NaCl, delaying seed germination and causing perturbations of both root and leaf development (Figure 5). The *rpotmp* mutant has reduced resistance to abiotic stresses (see [69] for light and drought stress). In contrast, complex I-deficient mutants are known for increased stress tolerance [40], so it is possible that the reduced content of complex IV causes *rpotmp* stress sensitivity. The absence of RPOTmp in mitochondria resulted in the suppression of hypoxia- and salicylate-responsive gene expressions (Figure 7), which possibly indicates compromised stress resistance. On the other hand, RPOTmp overexpression promoted seedling development under salt stress conditions, and the mitochondrial targeting of recombinant RPOTmp appeared to provide higher salt stress tolerance during the first two weeks of development (Figure 5). The germination test data suggest some differences in the ABA metabolism and/or perception between the studied transgenic lines and the WT.

### 3.2. Organellar Gene Expressions Are Affected by RPOTmp Overexpression

In agreement with earlier studies [27,30], our microarray analysis of transgenic lines with RPOTmp complementation in either mitochondria (Tmp-M3) or chloroplasts (Tmp-P1) additionally confirmed the specific role of RPOTmp in mitochondria (Figure 8 and Figure 9). While the *rpotmp* and the Tmp-P1 lines had almost identical mitochondrial transcript level profiles, the profile of the Tmp-M3 line was close to that of the wild type. The number of identified RPOTmp-dependent mitochondrial genes turned out to be more significant than previously shown [27], and this included several ORFs: the transcript levels changed in opposite directions in the *rpotmp* (reduced) and the OEM15 lines (increased), which proves that they are transcribed by RPOTmp (Figure 8 and Figure 9).

Most of the chloroplast genes were transcribed by PEP polymerase, and several by NEP polymerase [70,71,72]. The data of [27,30] and the present study show that the level of chloroplast transcripts remained unchanged in the *rpotmp* knockout line, except for the *rrn16* operon transcript initiated from the PC-promoter [29,30], which, however, did not affect the level of the mature *rrn16* transcript. The plants of the Tmp-M line were phenotypically almost indistinguishable from the wild-type plants, suggesting that RPOTmp is not essential in plastids for plant growth. An increase in the transcript levels of three NEP-dependent genes (*accD*, *clpP*, and *rpoB*) and the *rrn16* operon initiated from the PC-promoter was detected in the OEP lines (Figure 11), while the transcript levels of some other NEP-dependent genes (*rbcL*, *ycf3*) remained unaffected, assuming that RPOTmp has different affinity for diverse NEP promoters. These transcript level changes strongly suggest that the recombinant RPOTmp protein is present and active only in chloroplasts of the OEP lines, and it is not present in the chloroplasts of OEM lines. The increase in some transcript levels could trigger regulatory events leading to elevated seedling growth and development.

The expression of mitochondrially encoded genes in plants is mainly regulated post-transcriptionally [73]. A significant decrease in the expression of two mitochondrial editing factors (PPR proteins) in the OEM15 line (Data S1) indicates a possible connection between RPOTmp expression and the post-transcriptional regulation of genes encoding subunits of respiratory complexes. These two factors are MEF3, a sequence-specific RNA editing factor involved in the editing of the *atp4*-89 site in plant mitochondria [74], and the product of the AT3G15930 gene, which is co-expressed with the NAD5B transcript (ATMG00665) of complex I according to the STRING database. We hypothesize that the expression of these two proteins in the OEM15 line is suppressed in response to an excess of the primary mitochondrial transcripts accumulated in mitochondria, representing the negative regulation of mitochondrial gene expression.

### 3.3. Mitochondrial Retrograde Regulation Factor Expressions Are Affected by Rpotmp Knockout

Reduced transcript levels of several ETC complex I and IV subunits in the *rpotmp* mutant line decreased the content of assembled complexes and SCs, as well as potentially led to the imbalance and dysfunction of the ETC. The nucleus responds to mitochondrial dysfunction by switching on alternative respiration pathways, branched chain amino acid degradation, and the upregulation of several chaperones and transport proteins [75]. The changes in transcript levels of the nuclear-encoded genes observed in the *rpotmp* mutant line are most likely a general cellular response to retrograde signals of mitochondrial dysfunction and/or a more specific response to a deficiency of the mitochondrial respiratory complexes I and/or IV.

Using the rpotmp mutant, Merendino et al. [76] demonstrated the physiological impact of mitochondrial dysfunctions during skoto-morphogenesis (plant development in darkness), which consisted of transcriptomic alterations specifically dependent on the activity of the AOX1a enzyme, a key marker of mitochondrial retrograde regulation [75] that is induced in response to various mitochondrial and chloroplast perturbations [77]. However, in our experiments, *AOX* genes were not found among the DEGs of the studied lines. A significant increase in mitochondrial dysfunction [77] and MRR [36] marker transcript levels (*AT12CYS-2*, *UPOX*, and *HRG1)* was observed in *rpotmp* and complementation lines (Tmp-M3 and Tmp-P1) (Data S1).

Multiple upstream regulatory factors of MRR include transcription factors of the NAC family, as well as ANAC017 (activates ANAC013 transcription [55]) and ANAC013 (induction correlates with the ethylene response, ROS production, and AOX1a transcript level [78]). The ANAC013 transcript level was highly upregulated in both *rpotmp* and *ndufs4* lines, while ANAC017 was not (Figure 12). Overexpression of ANAC013 strongly induces *At12CYS-2* transcription [55] since ANAC013 binds to the promoters of genes encoding AOX1a [79] and At12CYS-2 [55]. A high expression of *AT12CYS-2* and *AOX1A* appeared to limit the plastid gene expression (PGE) in rifampicin-treated seedlings [80].

Van Aken et al. [81] showed that ANAC017 had little or no control over transcription within the mitochondrion, confirming reports [82] that nuclear and mitochondrial transcription are largely regulated independently. The absence of ANAC017 did not prevent the induction of AOX1A, At12CYS-2, and NDB4 by rifampicin treatment, indicating the existence of alternative retrograde pathways independent of canonical factors in seedlings subjected to mitochondrial stress in response to plastid dysfunction in the dark [80]. Also, as shown in [83], blocking ethylene signaling partially suppressed mitochondria-to-nucleus signaling independently of ANAC017. Our observations highlight the importance of At12CYS-2 and ANAC013 as regulatory factors signaling mitochondrial respiratory complex I dysfunction. As At12CYS proteins are located in both chloroplasts and mitochondria, they likely coordinate some of the functions that mediate interorganellar communication [20].

The *Arabidopsis* lines lacking RPOTmp in mitochondria were characterized by a significant increase in the expression of the membrane sensor protein TSPO (Data S1). *TSPO* transcript levels are known to be elevated in response to ABA, salt, and osmotic stress in seedlings [84]. Depending on the stress condition, TSPO was found in the membranes of mitochondria and ER [84]. Salt stress leads to a re-localization of the AtTSPO from ER to chloroplasts [85]. During salt and other stress conditions, AtTSPO may play a role in transporting tetrapyrrole intermediates [85], which are important in plastid retrograde signaling and tolerance to environmental stresses [86]. We observed both a significant increase in TSPO expression in the lines lacking RPOTmp in mitochondria and a significant deleterious effect of increased ABA and salt concentrations on their early development (Figure 4 and Figure 5 and Figure S3), indicating the possible involvement of TSPO in the cellular response to mitochondrial dysfunction in these lines.

### 3.4. Several TFs May Be Responsible for the Low Stress Sensitivity of RPOTmp Overexpressors

The decreased sensitivity of RPOTmp overexpressors to sucrose, ABA, and salt stress can be explained in the context of multifactorial events, where the altered activity of not only RPOTmp, but also other RPOTs is superimposed on altered hormonal metabolism. Bychkov et al. [68] showed that the hormone-related expression of the mitochondrial genome is at least, in part, regulated via the genes encoding RPOTmp and RPOTm, as well as the MTERF and SWIB family members. These genes were downregulated by ABA in the WT plants, modulating the accumulation of mitochondrial gene transcripts. The disruption of *RPOTmp* blocks or attenuates the hormone-dependent responses of mt-encoded genes, and it alters the sensitivity of the *rpotmp-2* mutant to hormone treatment. However, hormone-related changes in the transcriptional activity of mitochondrial genes may be modulated indirectly, suggesting that additional factors are needed for their regulation [68].

The expression of ABI4, one of the tetrapyrrole-signaling components that positively regulates plant development and the response to environmental stresses [86], was not changed in the RPOTmp overexpressors. It seems that other factors not associated with ABI4-dependent ABA degradation were involved in the accelerated germination and the reduced ABA sensitivity of the lines with RPOTmp overexpression.

Salt stress can trigger the ABA-dependent signaling pathway in plants: AtWRKY66 was shown to play a positive regulatory role in mediating salt tolerance through an ABA-dependent signaling pathway, affecting expression changes in the salt stress- and ABA-related genes [87]. Our analysis revealed an increased transcript level of ABF1 (AT1G49720) and a decreased transcript level of WRKY66 (AT1G80590) in the OEM15 line (Figure S11), indicating that RPOTmp overexpression affected the response to ABA and salinity.

It is interesting to note that, in the OEP12 line, chloroplast lipid metabolism might be affected not only by an increase in the expression of the plastid *accD* gene (Figure 11c) encoding a subunit of multimeric acetyl-CoA carboxylase [88,89], but also by increasing the expression of TF WRI4 (Data S1), which is known to activate the transcription of genes encoding chloroplast proteins involved in fatty acid biosynthesis during seed and flower development [90]. WRI4 is also upregulated by salt stress, and it is involved in activating the cuticular wax biosynthesis in *Arabidopsis* stems [91]. Increased *accD* transcription due to increased RPOTmp levels in chloroplasts may cause activation of *WRI4*, affecting plant growth.

The lines overexpressing RPOTmp were characterized by decreased expressions of several NAC transcription factors: ATAF1 (ANAC002), ANAC102 in all the lines studied, and ANAC055 in the OEP lines (Figure 12). ANAC055, ATAF1, and ANAC102 were induced by a long-term treatment with ABA and/or during age-dependent senescence [58]. Overexpression of these genes increased sensitivity to ABA in *Arabidopsis* [92,93]. Under ABA treatment, these SNAC-A genes regulate the expression of glyoxalase and thioredoxin [58], which are known as antioxidant compounds and are induced by abiotic stress conditions. ANAC102 and ATAF1 are also potential regulators of brassinosteroid (BR) catabolism and seedling photomorphogenesis [94,95]. ATAF1 is a positive regulator of ABA biosynthesis, directly regulating the abscisic acid biosynthetic gene *NCED3* [96] and integrating ABA- and H_2_O_2_-dependent signaling with senescence [97]. ANAC102 localizes to both chloroplasts and nucleus in *Arabidopsis.* In the chloroplasts, it specifically localizes with the nucleoids, where it interacts with the chloroplast RNA polymerases [98]. The overexpression of ANAC102 in chloroplasts leads to reduced chloroplast gene expression and chlorophyll content, indicating that this TF functions as a repressor in chloroplasts. The ANAC102 factor also plays a role in mediating the response to low oxygen stress (hypoxia) in germinating seedlings [99]. In our study, the OEM and the OEP lines were characterized both by the lower sensitivity of their seedling development to ABA treatment and by decreases in the transcript levels of *ATAF1* and *ANAC102* (Figure 12), suggesting their participation in ABA-signaling. The overexpression of RPOTmp in mitochondria also led to the suppression of a cytochrome P450 family protein, CYP720A1 (Data S1), which is involved in BR biosynthesis [100]. This is an interesting observation linking RPOTmp with ANAC102, ATAF1, and BR regulation.

According to Alshareef et al. [101], *ATAF1* co-expressed with *ANAC055* appears to have a common role working as a negative regulator in the thermomemory of *Arabidopsis* seedlings. ANAC055 is involved in the jasmonic acid-mediated signaling pathway [102].

## 4. Materials and Methods

### 4.1. Plant Material and Growth Conditions

The seeds of wild-type *A. thaliana* (L.) Heynh. ecotype Columbia (Col-0), the *ndufs4* knockout line (Sail596_E11) from the SAIL collection [103], the *rpotmp* knockout line (GABI_286E07) from the GABI-Kat collection [104], and the *abi4-1* knockout line were obtained from the *Arabidopsis* Biological Resource Center at Ohio State University (Columbus, OH, USA).

Seeds were sown in pots filled with a mixture of compost/vermiculite (1:1, *v*/*v*). After 3 days at 4 °C, the seedlings were grown at 22 °C in a KBW720 growth chamber (Binder, Germany) at a photon flux density of 150 μmol·m^−2^·s^−1^ and with a 16 h light/8 h dark photoperiod. For analysis of the early growth stages, seeds were germinated on Petri dishes. The seeds were sterilized in 80% (*v*/*v*) ethanol, 0.05% (*v*/*v*) Triton X-100 for 10 min, and were rinsed twice with sterile deionized water. The seeds were sown in Petri dishes containing 0.5X Murashige and Skoog salts (Sigma-Aldrich) and 0.8% (*w*/*v*) Phytagel (Sigma-Aldrich), and they were grown under the conditions described above. At least 30 plants of each line were used to estimate growth characteristics. To assess root elongation in the seedlings, Petri dishes were placed vertically facing the light source. Roots were measured by ruler. Germination as a quantitative indicator was calculated as a percentage of the total number of sown seeds.

To test the effect of abscisic acid on seed germination, Petri dishes with 0.5× Murashige and Skoog medium supplemented with 0.2 or 0.5 µM of ABA were used. To test the effect of NaCl, Petri dishes with 0.5× Murashige and Skoog medium supplemented with 75, 100, 125, 150, or 200 mM of NaCl were used. NaCl was added into the flasks with medium components and sterilized by boiling before being poured into the Petri dishes.

### 4.2. Obtaining Transgenic Arabidopsis Plants

To obtain transgenic lines with an overexpression of recombinant RNA polymerase RPOTmp, two genetic constructs described in [30] were used. In these constructs, a sequence of the catalytic part of the *Arabidopsis RPOTmp* gene cloned in the binary plasmid pBI121 [105] was combined with a sequence encoding the transit peptide of mitochondrial RNA polymerase RPOTm (mtTmp-pBI121) or with a sequence encoding the transit peptide of chloroplast RPOTp (ptTmp-pBI121). The genetic constructs were introduced into *A. tumefaciens* according to [106]. The transformation of *Arabidopsis* with *Agrobacterium* was by the floral dip method [106]. As a result, homozygous lines with RPOTmp overexpression in mitochondria (OEM) or in chloroplasts (OEP) were obtained on the basis of wild-type plants. Lines with a complementation of RPOTmp functions in mitochondria (Tmp-M) or chloroplasts (Tmp-P) on the basis of *rpotmp* mutant were obtained as described in [30].

### 4.3. Microarray and Bioinformatic Analysis

Total RNA was extracted from the rosette leaves of 12-day-old plants using a Qiagen RNeasy plant mini kit (Qiagen, Hilden, Germany). cDNA was synthesized in AffinityScript-RT using random primers (Agilent Technologies, Santa Clara, CA, USA). cRNA samples were labeled with a two-color Low Input Quick Amp labeling kit and a two-color Agilent RNA Spike-In kit according to the manufacturer (Agilent Technologies) protocol. The cRNA samples of the Col-0 and transgenic lines were labeled with Cy3 and Cy5 fluorescent dyes, respectively, and purified. Microarray analysis was performed by hybridizing four arrays in a single *Arabidopsis* (V4) Gene Expression Microarray slide (4 × 44 K; Agilent Technologies) with a mixture of the Cy3- and Cy5-labeled cDNA. After hybridization by a Gene Expression Hybridization kit (Agilent Technologies), the microarray slide was scanned using a scanner model G2539A with scan control A.8.5.1 (Agilent Technologies). Data analysis was performed using Feature Extraction 10.10.1.1 (Agilent Technologies). For the analysis, RDyeNormSignal and GDyeNormSignal were used. The data processing was performed using the R programming language [107], the limma package [108], and Bioconductor.org [109]. Genes with expression value fold changes greater than 2, and had moderated *t*-test *p*-values (adjusted via the BH (Benjamini–Hochberg) method) of less than 0.2, were considered differential expressed genes (DEGs). The heat maps were drawn using the ComplexHeatmap package [110]. Plots were generated using ggplot2 package [111]. Gene set enrichment analysis was performed using ClusterProfiler package [112]. Venn diagrams were drawn using the Venn package [113].

### 4.4. Analysis of Gene Expressions and Copy Numbers

RNA was extracted from the seedlings using TRI Reagent (Sigma-Aldrich, St. Louis, MO, USA) in accordance with the manufacturer’s instructions, then treated with RNase-free DNase (Thermo Fisher Scientific, Waltham, MA, USA) and quantified with a NanoPhotometer NP80 (IMPLEN, Munich, Germany). The RNA quality was checked by electrophoresis in 1% (*w*/*v*) agarose gel. cDNA was synthesized using random hexamer primers and M-MuLV Reverse transcriptase (Thermo Scientific). Quantitative PCR (qPCR) amplification of cDNAs was performed with the SYBR Select Master Mix (Applied Biosystems, Waltham, MA, USA) and a CFX96 cycler (Bio-Rad, Hercules, CA, USA). All reactions were performed in duplicate. The expression of each gene was normalized using the expressions of the *YLS8* (at5g08290) and *AP2M* (at5g46630) genes [114]. RT-qPCR analysis was repeated three times with independently isolated RNA samples. Primer sequences are listed in Supporting Table S1.

The DNeasy Plant Mini kit (Qiagen) was used for DNA isolation in accordance with the manufacturer’s instructions. The gene copy number analysis was performed with qPCR as described above employing the primers used for RT-qPCR. The nuclear *ACT7* gene was used for normalization of the data (Table S1).

### 4.5. BN-PAGE and In-Gel Activity Staining

Mitochondria were isolated from the 3-week-old plants of *A. thaliana* in accordance with Sweetlove et al. [115] with modifications. Mitochondrial membranes (100 µg of protein) were solubilized in 30 µL of a buffer containing 30 mM of HEPES, 150 mM of K-acetate, 10% glycerol, 2 mM of PMSF, and 5 mg of digitonin per 1 mg of protein. The supernatant enriched with the mitochondrial membrane complexes was obtained after centrifugation at 14,200× *g* (30 min) and then mixed with a 5% (*w*/*v*) solution of Coomassie brilliant blue G-250.

Blue native electrophoresis in polyacrylamide gel (BN-PAGE) was performed in protein electrophoresis chambers with glass sizes of 16 × 20 cm (Bio rad Protean II xi) in accordance with [116]. BN gradient gels with concentrations of 3.5–13% acrylamide were used. Electrophoresis conditions were 105–110 V overnight. The 2D BN/SDS-PAGE was carried out in a protein electrophoresis chamber with a glass size of 15 × 15 cm (Helicon, Moscow, Russia) according to the method described in [117]. A 2D BN/SDS gradient gel with concentrations of 10–20% acrylamide was used. The electrophoresis conditions were 4 °C and 150 V overnight. The gel was stained with colloidal Coomassie (8% (NH_4_)_2_SO_4_, 1.6% H_3_PO_4_, and 0.08% Coomassie G-250, 20% EtOH).

The in-gel activity staining of complexes I and IV in the BN-PAGE gel was performed according to established protocols [118,119]. The activity of complex I was evaluated by gel incubation in a buffer containing 0.1 M of Tris-HCl, 0.225 mM of NADH, and 157 µg/mL of nitroblue tetrazolium (NBT) for 10 min. Activity of complex IV was evaluated by gel incubation in a buffer containing 50 mm of KH_2_PO_4_, 0.1% diaminobenzidine, 1 mg/mL of cytochrome c, 7.5% sucrose, a pH of 7.4 for 60 min.

### 4.6. Western Blotting

Transfer of the gel obtained by 2D BN/SDS-PAGE to a nitrocellulose membrane (Bio-Rad Transblot transfer medium) was performed using a buffer (48 mM of Tris, 35 mM pf glycine, 10% methanol, and a pH of 9.2) and a Bio-Rad Mini Trans-blot Cell chamber for 3 h at 4 °C at a constant current of 400 mA. The membrane was incubated overnight at 4 °C in a blocking solution containing 200 mM of Tris, 500 mM of NaCl, 53 mM of NaN_3_, 5% (*w*/*v*) skimmed milk powder, and a pH of 7.5. Hybridization dilutions of the antibodies were prepared using the blocking solution. Commercial antibodies for *Arabidopsis* mitochondrial respiratory chain proteins (75 kDa, SDH1-1, CYC1, *Nad9*, *CoB*, and ATP3) were obtained from PhytoAB Inc. (San Francisco, CA, USA) and used in a dilution ratio of 1:2000. The used antibodies are listed in Table S2.

### 4.7. Statistical Analysis

The experiments were repeated three times or more. All experimental data were statistically analyzed using parametric (Student’s *t*-Test) and non-parametric tests (Kruskal–Wallis test and Mann–Whitney test). Verification of the distribution normality was carried out using the Shapiro–Wilk test. Mean values ± standard deviations of the mean (SD) were calculated from the results of at least three replicates, and significant differences relative to the controls are presented at * *p* < 0.05, ** *p* < 0.01, *** *p* < 0.001, and **** *p* < 0.0001.

## 5. Conclusions

We have shown that an increase in the content of RPOTmp in mitochondria or chloroplasts leads to an increase in the level of transcripts of several mitochondrial (*nad4*, *cox1*, *matR*, etc.) or chloroplast (*accD*, *clpP*, and *rpoB*) genes, respectively. The accumulation of organellar transcripts somehow triggers a complex signaling cascade leading to faster growth, early flowering, lowered ABA sensitivity, and an increased stress resistance in the RPOTmp-overexpressing plants. Our work shows for the first time that the overexpression of RNA polymerase targeting mitochondria or chloroplasts induces an intensive retrograde response. Despite the similarity of the phenotype of the OEM and the OEP plants, the responses at the transcriptome level turned out to be highly specific to mitochondrial or chloroplast localization of overexpressed RPOTmp. Although the exact nature of the signal from the organelles to the nucleus remains unclear, NAC family transcription factors are involved in downstream regulatory events. ANAC013 and ANAC055 probably take part in signal transduction from mitochondria and chloroplasts, respectively, while ATAF1 and ANAC102 seem to participate in signaling from both organelles. A decreased sensitivity of the OE plants to ABA could also be at least, in part, modulated by the TFs of the NAC family. Many other factors, including TFs from different families and PPR-proteins, could also take part in the re-orchestration of metabolism in response to overexpression/absence of RPOTmp in organelles.

## Data Availability

The access of mRNA expression profiling data and the primary data of the mRNA profiles of the rpotmp mutant lines overexpressing or complementing RPOTmp in mitochondria (OEM15 and Tmp-M3) or in chloroplasts (OEP12 and Tmp-P1) are available at the Gene Expression Omnibus (GEO) site (http://www.ncbi.nlm.nih.gov/geo/, accessed on 26 December 2023). Accession number GSE251661 (https://www.ncbi.nlm.nih.gov/geo/query/acc.cgi?acc=GSE251661, accessed on 26 December 2023).

## Supplementary Materials

The following supporting information can be downloaded at: https://www.mdpi.com/article/10.3390/ijms25158164/s1.
