# Supplementary material for: Overexpression of RPOTmp Being Targeted to Either Mitochondria or Chloroplasts in Arabidopsis Leads to Overall Transcriptome Changes and Faster Growth
Source: Int J Mol Sci. 2024 Jul 26;25(15):8164. doi: 10.3390/ijms25158164 (PMC11312007; doi:10.3390/ijms25158164)
Supplement: Supplementary file 1 [file ijms-25-08164-s001.zip › Supporting information edited.pdf]

Supporting information

Table S1. Primers used for quantitative RT-PCR analysis.

Gene/transcript name	Forward primer	Reverse primer
rpoT2	GCGTTGGTTTGGTGAATGTGC	AAGCGATAGGGTCTGGAGGGA
cox1	AGTCATTCTGGAGGAGCAGTTGA	ACGGACCACACAAATAGGGGTAA
nad3	ACCTTTTTCTTTCCTTGGGCAGT	CGAAGCACCCCTTTTCCATTCA
nad4	GCATTTCAGTGGGTTGGTCTGGT	AGGGATTGGCACGCTTTCGG
nad6	AGCCGTTTCATTCCTTTTCGTTG	GAGGTCGTATTTCTTTGGGTTGGT
accD	GCCCTTGGGGTTATGGATTTTCG	TCCTTCTTGCATTCGTGCTCCT
clpP	AACTCTCCTGGTGGATGGGT	ATTCTCCCGTTTGTGCCTCATAA
petB	TGACTTTTTATTACCGTCCAACCGT	ACCATCATACTTGCCGACCATCG
psbA	CGGTGCCATTATTCCTACTTCTGC	AGTTCCCACTCACGACCCAT
rbcL	TGCCGTAGCCAACCGAGT	TCTTTCCATACTTCACAAGCAGCA
rpoB	GGCAGCGGGTAGGCGAAAT	CGGAGCATCTTCTGGTTTAGGT
rrn16	AAGTCCGCCGTCAAATCCCA	AGTGCTTTCGCCGTTGGTGT
ycf3	ACGAAACCCCTTTTTACCACAAGC	CGAACCAAGCCTCCGCCAT
yls8	GAGGTGCTTGCGTCTGTTGCT	TGTCCTTGAGAGCCCAGTTGAT
clathrin	GCTGTTGGTTGGAGAAGAGAGG	TCCCCGTCACATCACACCGA
act7	GCACCGCCAGAGAGAAAATACAG	ACCACCACGAACCAGATAAGACA
P2-initiated RNA	AGCGAACTCCATGCGAATATGAA	GCCAGGATCGAACTCTCCATGA
PC-initiated RNA	GGGTAGCTATATTTCTGGGAGCGAA	GCCAGGATCGAACTCTCCATGA
upstream-initiated RNA	GGCTCGTGGGATTGACGTGAG	GCCAGGATCGAACTCTCCATGA

Primer sequences are given in 5’- 3’ orientation.

Table S2. The list of antibodies obtained from PhytoAB Inc (USA) and used in the current study.

S/N	Subunit	ETC Complex	TAIR ID
PHY0523S	75 kDa	I	AT5G37510
PHY1408S	SDH1-1	II	AT5G66760
PHY0566S	CYC1	III	AT5G40810
PHY0578S	COXX6	IV	AT2G16460
PHY0595A	ATP3	V	AT2G33040
PHY1080A	NAD9	I	ATMG00070
PHY1116A	COB	III	ATMG00220

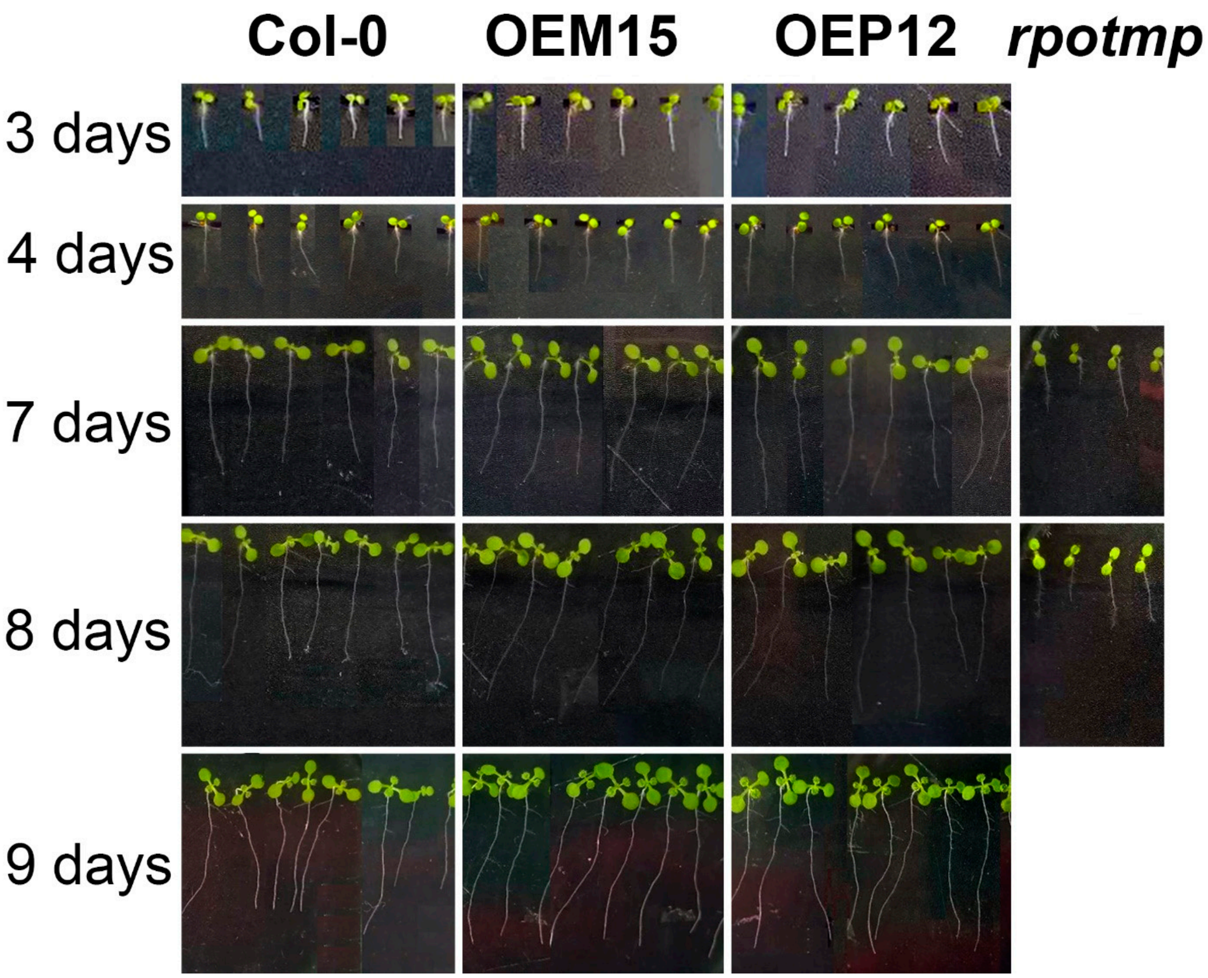

Figure S1. Phenotypic features of Arabidopsis lines with overexpression of RPOTmp targeted to mitochondria (OEM) or chloroplasts (OEP). Primary roots of the wild-type line (Col-0), rpotmp mutant, OEM15 and OEP12 lines grown vertically on plates with a nutrient medium.

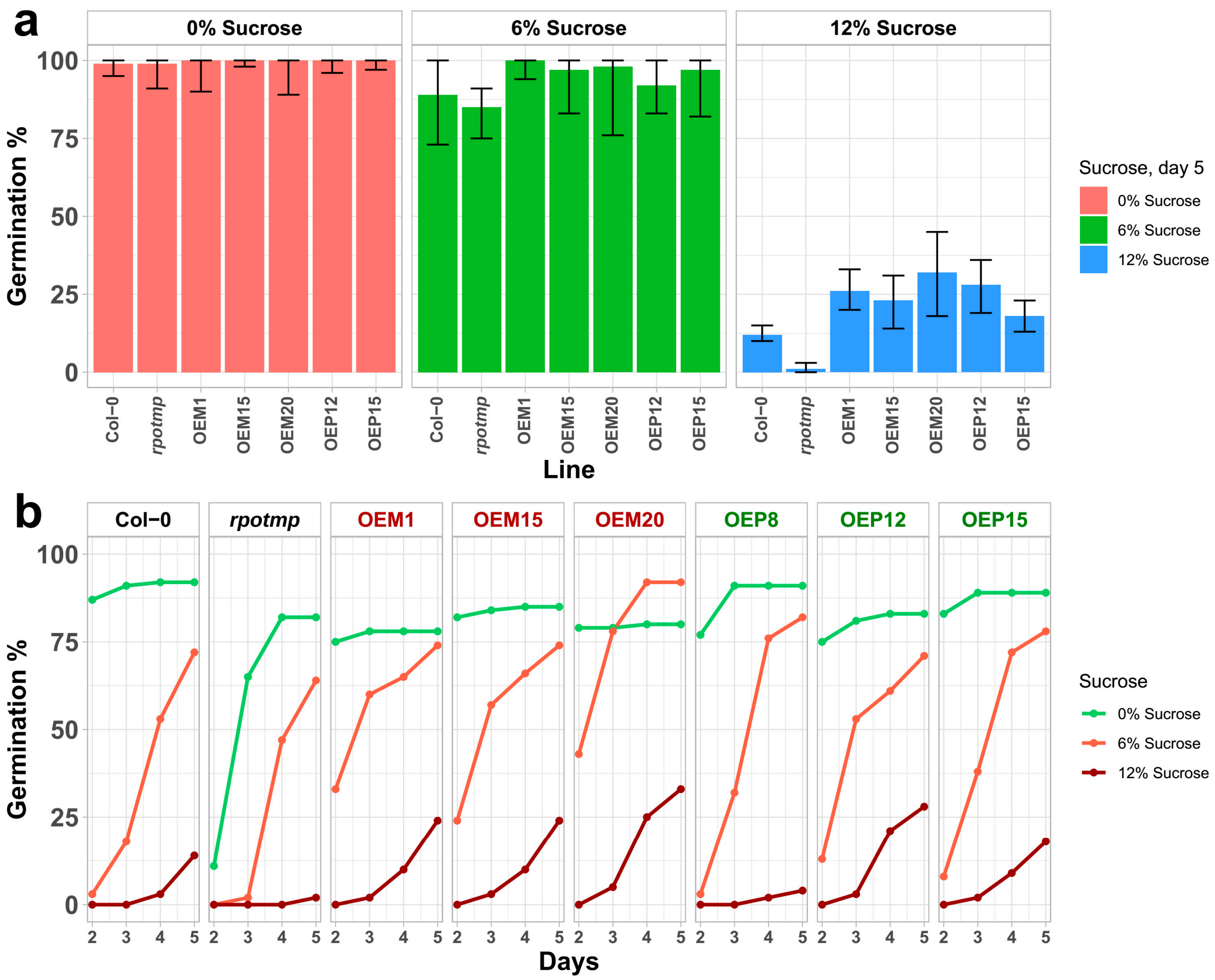

Figure S2. Germination of *Arabidopsis* lines with altered RPOTmp expression on the growth media containing elevated amounts of sucrose.

(**a**) Seed germination on growth media containing 0%, 6% or 12% sucrose, day 5 after the end of stratification. The graph shows the results of two independent experiments, number of seeds of each genotype on each growth medium in one experiment: n = 150.

(**b**) Seed germination rate on growth media containing 0%, 6% or 12% sucrose for 2-5 days. The graph shows the results of one experiment, the number of seeds of each genotype on each nutrient medium in one experiment: n = 80.

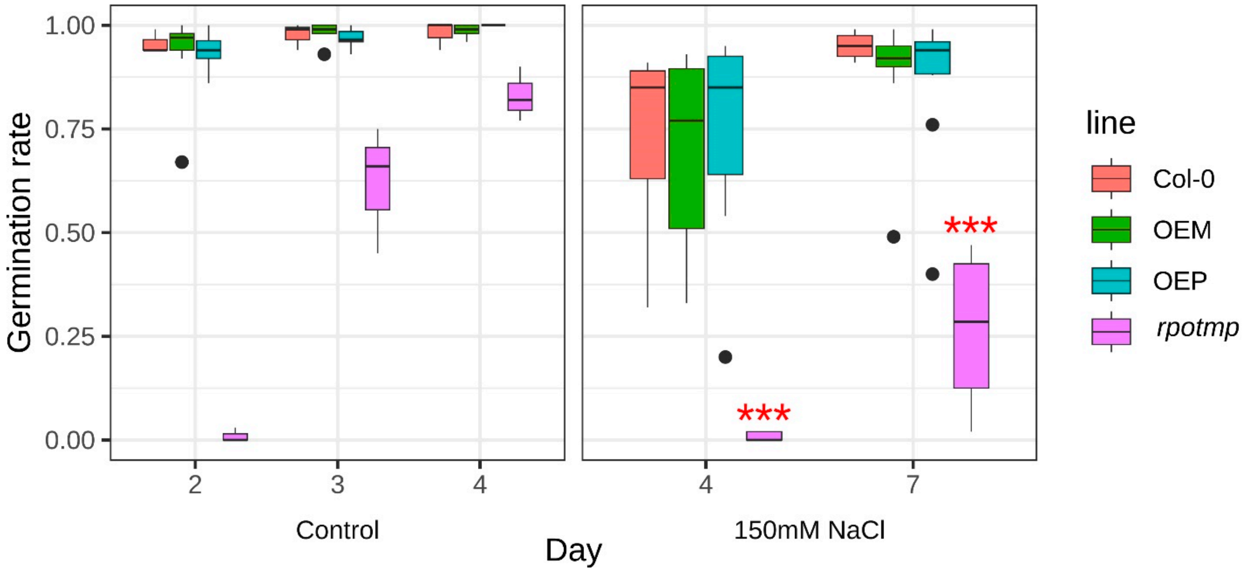

Figure S3. Germination of *Arabidopsis* lines with altered RPOTmp expression on the growth media containing NaCl.

Seeds of *Arabidopsis* lines with altered RPOTmp expression (*rpotmp*, OEM and OEP lines) were germinated on the growth media without (control) or with 150 mM NaCl. The germination rate was estimated on day 2, 3, 4 and 7 after the end of stratification. The boxes show the results of two independent experiments, number of seeds of each genotype on each growth medium in one experiment: n = 50. Asterisks indicate differences from Col-0: ***—$p < 0.001$ (Mann-Whitney-Wilcoxon test).

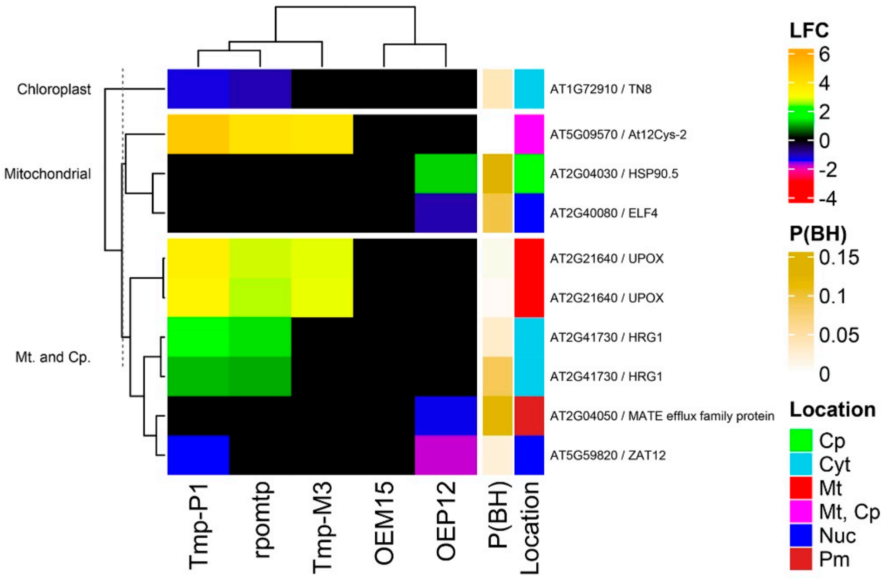

Figure S4. Differential expression of marker genes for mitochondria and chloroplasts perturbations in studied lines.

Heatmap of nuclear genes encoding marker factors in knockout mutant *rpotmp* and in lines with RPOTmp overexpression (OEM15 and OEP12) and RPOTmp function complementation (Tmp-M3 and Tmp-P1) is presented. The color key representing LFC-to-color transversion is provided. P(BH) stands for multiple comparison F-test p-values adjusted by Benjamini-Hochberg method. Location shows the cellular localization of the proteins: Cp – chloroplast, Cyt – cytoplasm, Mt – mitochondria, Mt, Cp – dual mitochondrial-chloroplast localization, Nuc – nucleus, Pm – plasma membrane.

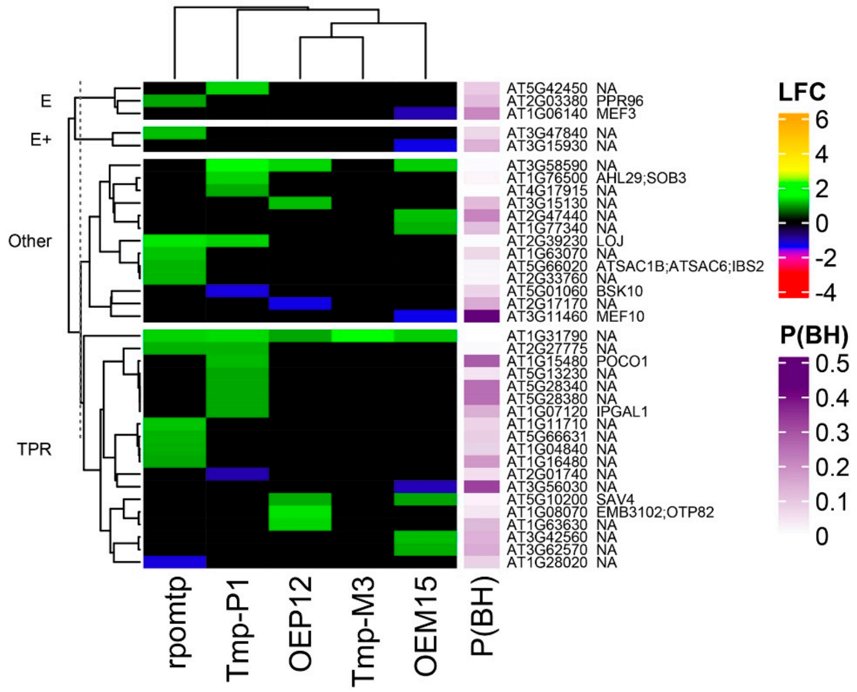

Figure S5. Expression of proteins with a tricopeptide repeat in stidued lines.
Heatmap of nuclear genes encoding proteins with a tricopeptide repeat in knockout mutant *rpotmp* and in lines with RPOTmp overexpression (OEM15 and OEP12) and RPOTmp function complementation (Tmp-M3 and Tmp-P1) is presented. The color key representing LFC-to-color transversion is provided. P(BH) stands for multiple comparison F-test p-values adjusted by Benjamini-Hochberg method.

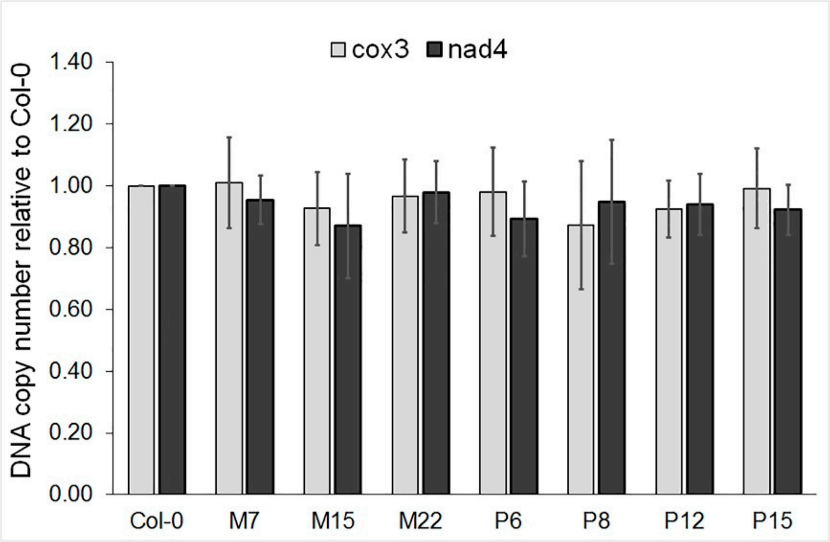

Figure S6. Copy number of mitochondrial genes in studied lines.
Analysis of the copy number of two mitochondrial genes, *COX1* and *NAD3*, was performed using the real-time PCR. Data from three independent biological replicates are presented. The *ACT7* nuclear gene was used for normalization. The transcript level in Col-0 was taken as a unit.

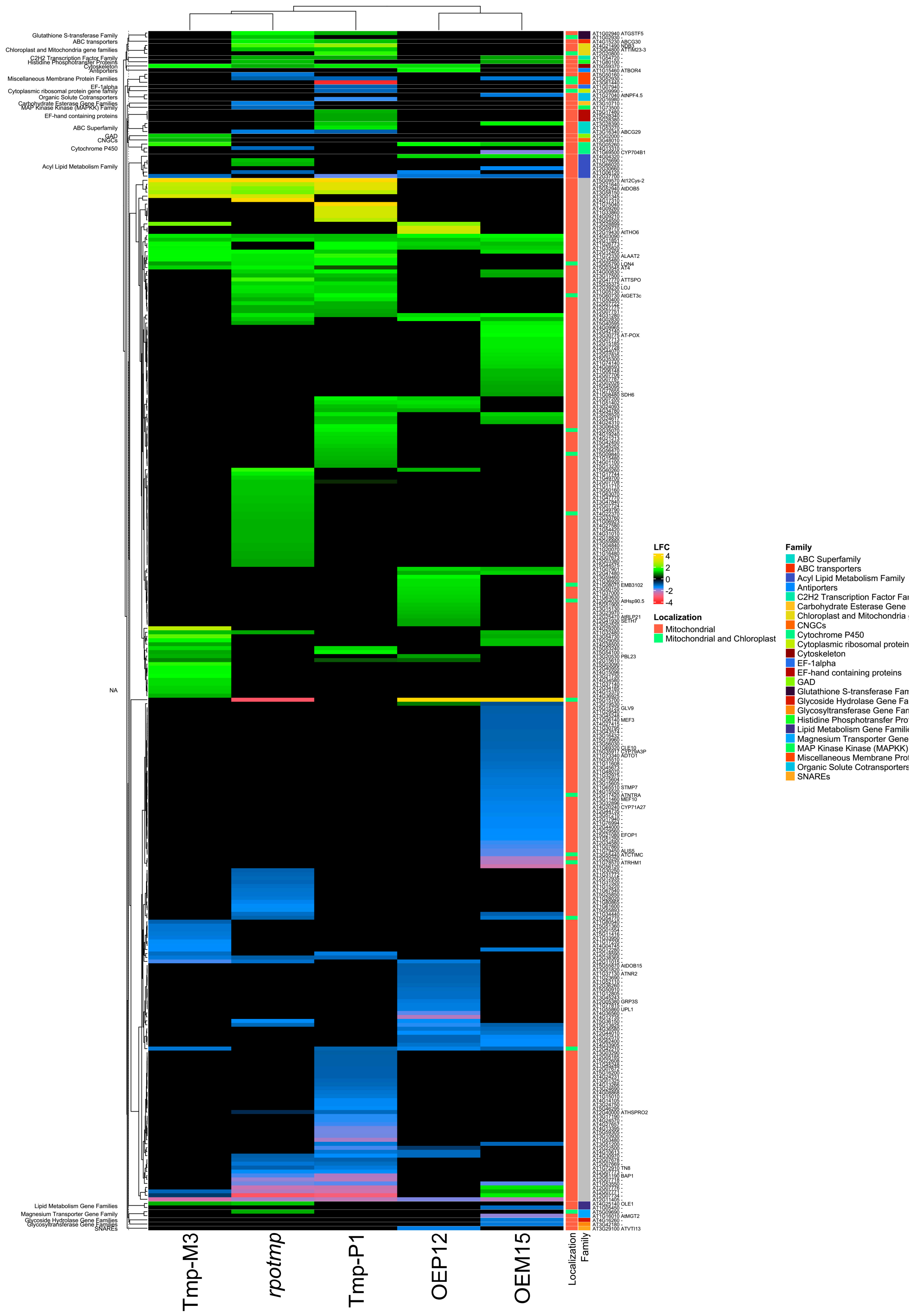

Figure S7. Differential expression of nuclear genes encoding mitochondrial proteins in studied lines.

Heatmap of mitochondrial proteins transcripts encoded by the nuclear genes in knockout mutant *rpotmp* and in lines with RPOTmp overexpression (OEM15 and OEP12) and RPOTmp function complementation (Tmp-M3 and Tmp-P1) is presented. The color key representing LFC-to-color transversion is provided. Right side annotation represents protein localization, and gene families.

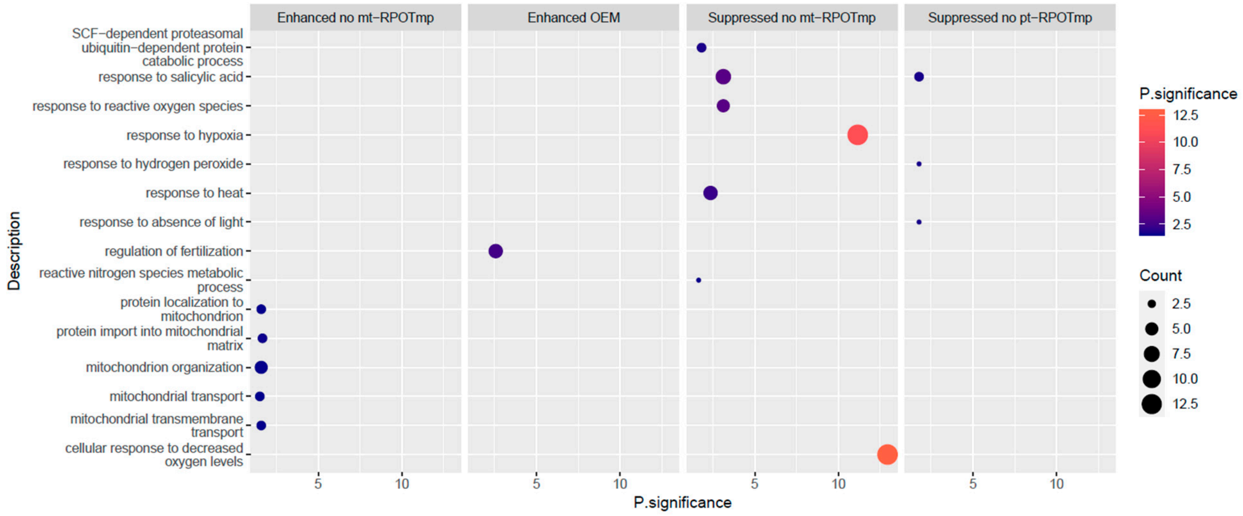

Figure S8. Go terms enrichment analysis of genes identified as possible retrograde regulation response to altered amount of RPOTmp in organelles. “Enhanced no mt-RPOTmp” and “Suppressed no mt-RPOTmp” stands for enhanced and suppressed DEGs of both *rpotmp* mutant and Tmp-P1 that were absent from DEGs of OEM15 and OEP12. “Enhanced OEM” stands for enhanced DEGs of OEM15 that are absent from DEGs of OEP12, Tmp-P1 and *rpotmp.* “Suppressed no pt-RPOTmp” stands for suppressed DEGs of both Tmp-M3 and *rpotmp* that were absent from DEGs of OEM15 and OEP12.

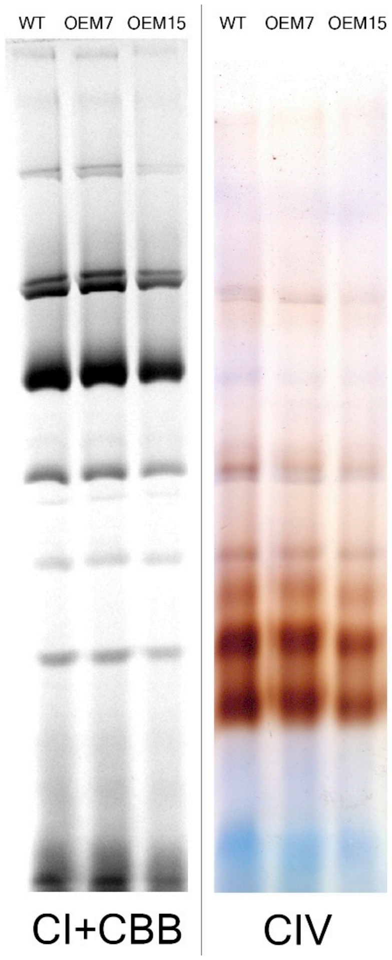

Fig. S9. Activity of respiratory complex IV in the OEM lines.
BN-PAGE electropherograms of protein complexes solubilized from the mitochondrial membranes of the wild type (C), OEM7 and OEM15 lines. Protein complexes were stained using complex IV activity staining (CIV) or by CBB (Coomassie Brilliant Blue) after complex I activity staining (CI+CBB).

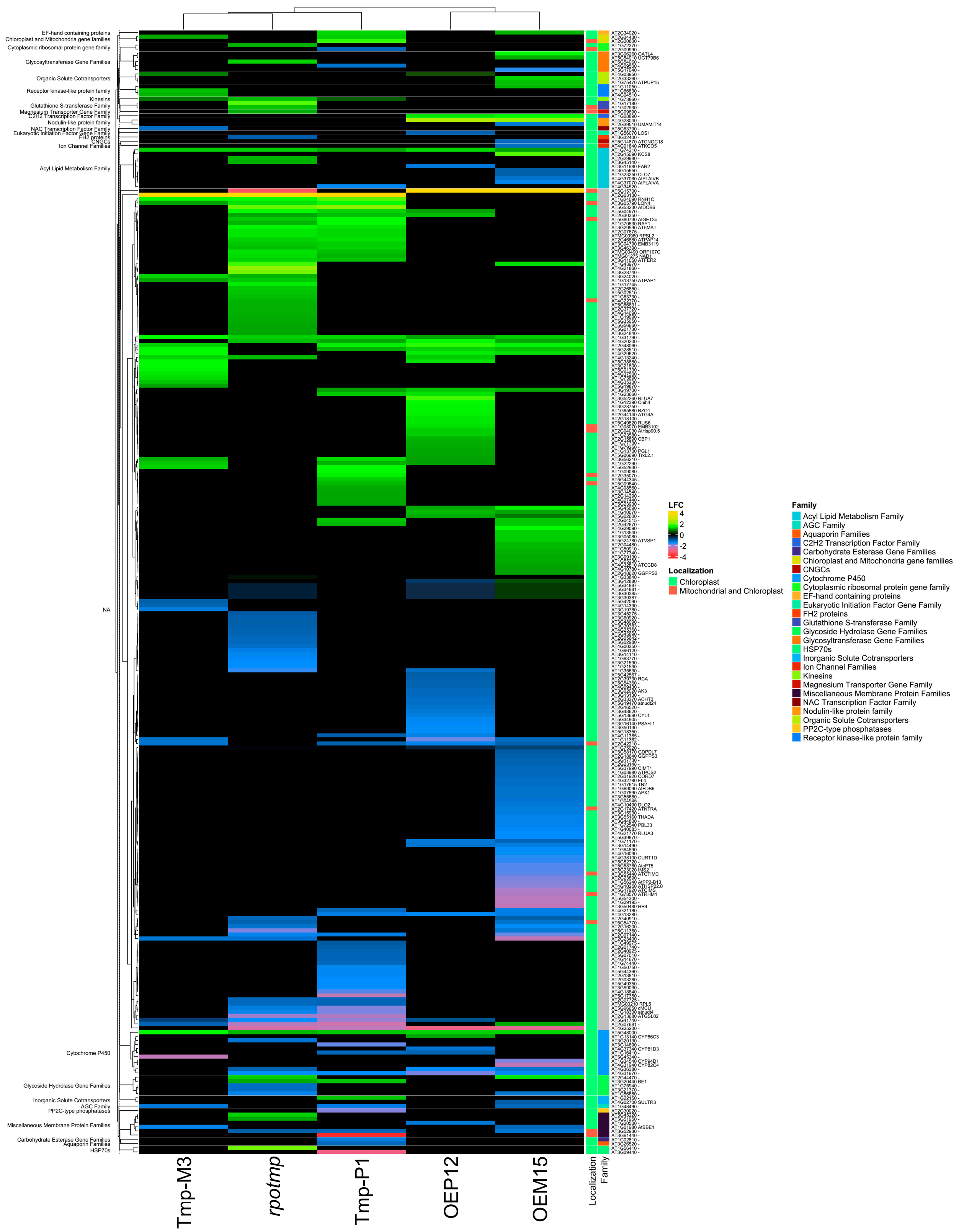

Figure S10. Differential expression of nuclear genes encoding chloroplast proteins in studied lines.

Heatmap of chloroplasts proteins transcripts encoded by the nuclear genes in knockout mutant *rpotmp* and in lines with RPOTmp overexpression (OEM15 and OEP12) and RPOTmp function

complementation (Tmp-M3 and Tmp-P1) is presented. The color key representing LFC-to-color transversion is provided. Right side annotation represents protein localization, and gene families.

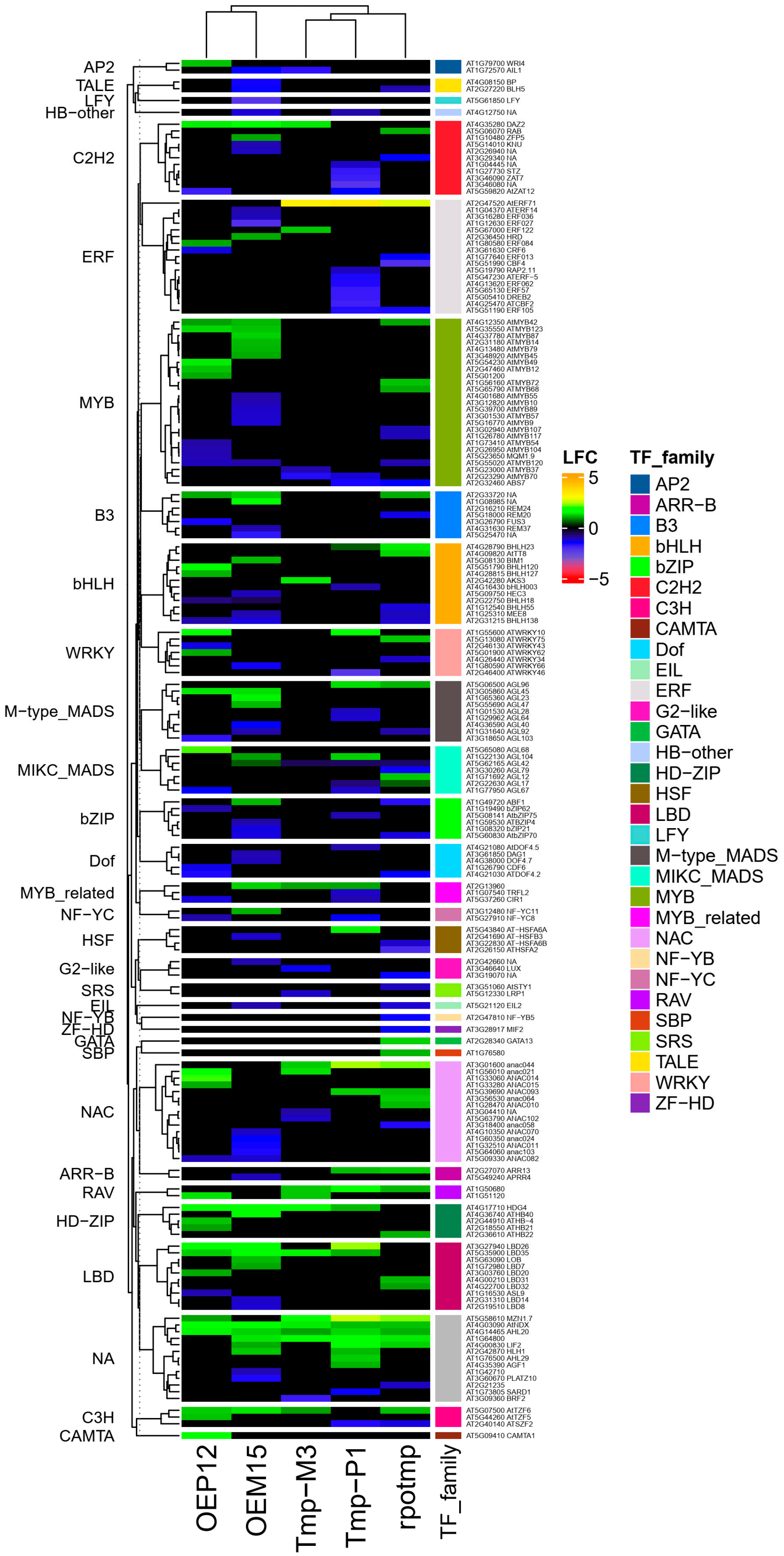

AP2
TALE
LFY
HB−other
C2H2
ERF
MYB
B3
bHLH
WRKY
M−type_MADS
MIKC_MADS
bZIP
Dof
MYB_related
NF−YC
HSF
G2−like
SRS
EIL
NF−YB
ZF−HD
GATA
SBP
NAC
ARR−B
RAV
HD−ZIP
LBD
NA
C3H
CAMTA
OEP12
OEM15
Tmp−M3
Tmp−P1
rpotmp
TF_family
LFC
5
0
−5
TF_family
AP2
ARR−B
B3
bHLH
bZIP
C2H2
C3H
CAMTA
Dof
EIL
ERF
G2−like
GATA
HB−other
HD−ZIP
HSF
LBD
LFY
M−type_MADS
MIKC_MADS
MYB
MYB_related
NAC
NF−YB
NF−YC
RAV
SBP
SRS
TALE
WRKY
ZF−HD

Figure S11. Differential expression of nuclear genes encoding transcription factors in studied lines.
Heatmap of nuclear genes encoding transcription factors in knockout mutant *rpotmp* and in lines with RPOTmp overexpression (OEM15 and OEP12) and RPOTmp function complementation (Tmp-M3 and Tmp-P1) is presented. The color key representing LFC-to-color transversion is provided. Right side annotation represents transcription factors family.
